# Efficient solid-phase synthesis and structural characterization of segetalins A–H, J and K

**DOI:** 10.3762/bjoc.21.202

**Published:** 2025-11-27

**Authors:** Liangyu Liu, Wanqiu Lu, Quanping Guo, Zhaoqing Xu

**Affiliations:** 1 Key Laboratory of Preclinical Study for New Drugs of Gansu Province, School of Basic Medical Sciences, Lanzhou University, 199 West Donggang Road, Lanzhou 730000, China

**Keywords:** Fmoc-solid-phase peptide synthesis (Fmoc-SPPS), head-to-tail cyclization, plant cyclopeptides, *Vaccaria segetalis*

## Abstract

This study establishes an efficient solid-phase strategy for the total synthesis of segetalins A–H, J and K (**1**–**10**), bioactive cyclopeptides isolated from *Vaccaria segetalis*. Linear precursors were assembled on cost-effective 2-chlorotrityl chloride resin via Fmoc-SPPS, followed by PyBOP-mediated head-to-tail cyclization in DMF (10^−3^ M). After RP-HPLC purification, all cyclopeptides were obtained in 45–70% isolated yields. Structural identities were confirmed by HRESIMS, NMR, and HPLC (>95% purity). Circular dichroism (CD) spectroscopy revealed distinct secondary structures, including β-sheets (**1**, **2**, **3**, **4**, **7**, **8**, **10**) and α-helical elements (**5**, **6**). This scalable methodology overcomes limitations of prior syntheses, enabling biological evaluation.

## Introduction

Cyclopeptides have garnered significant research interest owing to their unique conformational constraints imposed by cyclization and diverse biological activities [1–3]. Specifically, plant-derived cyclopeptides represent a valuable source of potential lead compounds for drug discovery [4]. Segetalins A–H, J and K (**1**–**10**), isolated from the seeds of *Vaccaria segetalis* (Caryophyllaceae), are head-to-tail cyclic oligopeptides comprising 5–9 amino acid residues [5–13]. These natural products exhibit a significant diversity of pharmacological activities [14–16], including estrogen-like activity (**1**, **2**, **7**, **8**), antitumor effects (**5**), and antimicrobial properties (**3**). Given their unique structural features and pharmacological potential, segetalins have become important targets for both synthetic chemistry and drug development. However, efficient and general synthetic routes to access this family have remained limited over the past decades.

Previous synthetic approaches have encountered significant challenges. Sonnet et al. reported the first total synthesis of segetalin A (**1**) via Sasrin resin-based SPPS, followed by cyclization under highly dilute conditions (10^−4^ M) with diphenylphosphoryl azide (DPPA) [17]. While successful, this approach suffers from the high cost of the specialized resin and large solvent volumes required for dilution, coupled with DPPA's poor efficiency in forming sterically hindered peptide bonds involving residues like Val or Ile. Dahiya and Kaur synthesized segetalin C (**3**) via a solution-phase fragment coupling strategy, culminating in cyclization mediated by *N*,*N*'-dicyclohexylcarbodiimide (DCC)/*N*-methylmorpholine (NMM) at 0 °C for 7 days [18]. This method, however, is lengthy, operationally complex, difficult for product isolation, and carries a significant risk of racemization. Wong and Jolliffe synthesized segetalins B (**2**) and G (**7**) using a pseudoprolinic acid strategy to induce *cis*-amide bond formation, followed by desulfurization [19]. Despite achieving cyclization, this route involves intricate procedures, expensive starting materials, and has limited applicability to other segetalins.

Given the limitations of existing methodologies and the biological significance of the segetalins, we sought to develop an efficient, scalable, and generally applicable solid-phase synthesis strategy for the *Vaccaria segetalis* cyclopeptide family.

## Results and Discussion

### Synthesis strategy and optimization

**Scheme 1:**
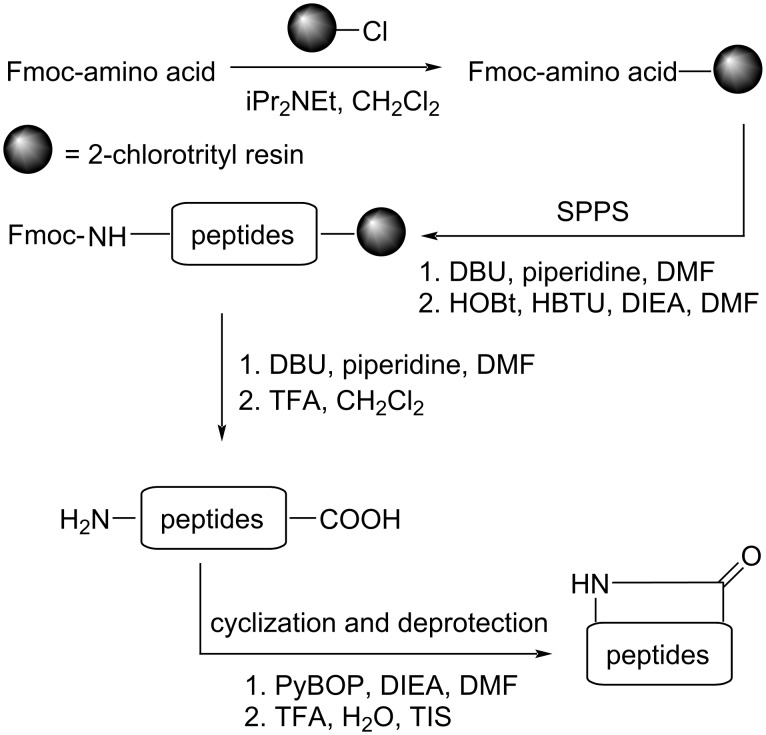
Preparation of segetalins A–H, J and K.

While both solution-phase and solid-phase approaches are viable for peptide synthesis, Fmoc-based SPPS offers distinct advantages in operational simplicity and efficiency for laboratory-scale production [20]. We therefore devised a streamlined solid-phase strategy for synthesizing the *Vaccaria segetalis* cyclopeptide family (Scheme 1).

Building upon previous work [17–19], we focused on optimizing key parameters: resin selection, Fmoc deprotection conditions, coupling reagents for linear assembly, and crucially, the cyclization step. Cost-effectiveness and commercial availability led us to select 2-chlorotrityl chloride resin as the solid support, enabling mild cleavage of the partially protected linear peptide precursor [21]. Efficient Fmoc deprotection was achieved using a solution of 1% pyridine and 1% 1,8-diazabicyclo[5.4.0]undec-7-ene (DBU) in *N*,*N*-dimethylformamide (DMF) [22]. For the assembly of the linear sequences, coupling efficiency was significantly enhanced using a 1:1 mixture of 1-hydroxybenzotriazole (HOBt) and 2-(1*H*-benzotriazol-1-yl)-1,1,3,3-tetramethyluronium hexafluorophosphate (HBTU) in DMF [23]. Finally, we obtained crude linear peptides with 75% to 95% yields (Table 1).

**Table 1:** Preparation of linear peptides for segetalins A–H, J and K.

	Structure	Yield^a^

1	Gly-Val-Pro-Val-Trp(Boc)-Ala	89%
2	Gly-Val-Ala-Trp(Boc)-Ala	77%
3	Gly-Leu-His(Trt)-Phe-Ala-Phe-Pro	93%
4	Gly-Leu-Ser(*t*-Bu)-Phe-Ala-Phe-Pro	91%
5	Gly-Tyr(*t*-Bu)-Val-Pro-Leu-Trp(Boc)-Pro	89%
6	Ala-Ser(*t*-Bu)-Tyr(*t*-Bu)-Ser(*t*-Bu)-Ser(*t*-Bu)-Lys(Boc)-Pro-Phe-Ser(*t*-Bu)	87%
7	Gly-Val-Lys(Boc)-Tyr(*t*-Bu)-Ala	95%
8	Gly-Tyr(*t*-Bu)-Arg(Pbf)-Phe-Ser(*t*-Bu)	94%
9	Phe-Gly-Thr(*t*-Bu)-His(Trt)-Gly-Leu-Pro-Ala-Pro	89%
10	Gly-Arg(Pbf)-Val-Lys(Boc)-Ala	87%

^a^Yield of crude linear peptide.

**Figure 1:**
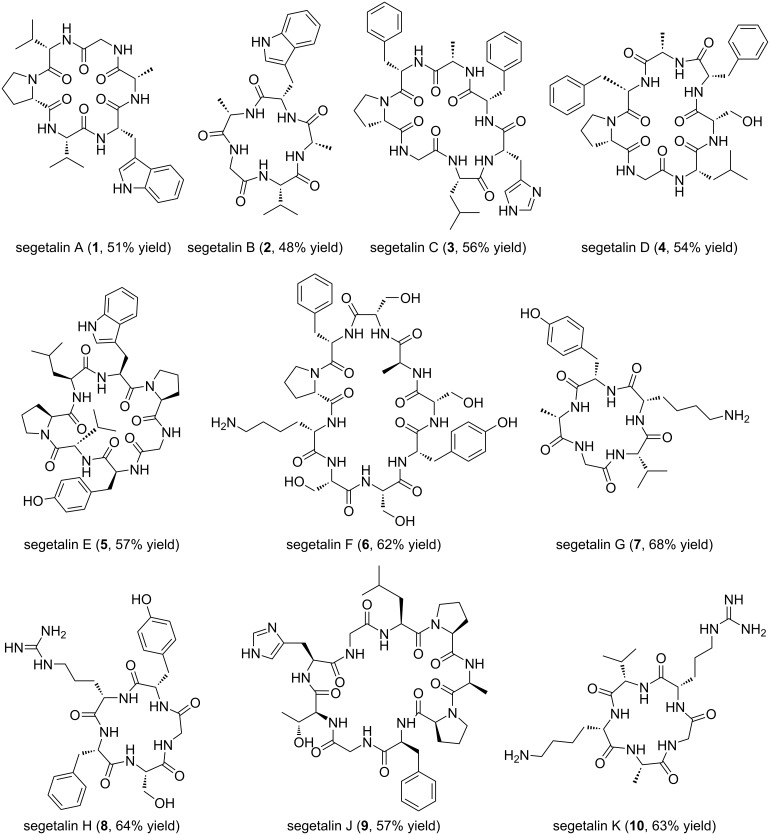
Cyclization reactions to segetalins A–H, J and K.

The critical head-to-tail cyclization step proved challenging. Initial attempts using common coupling reagents such as 1-[bis(dimethylamino)methylene]-1*H*-1,2,3-triazolo[4,5-*b*]pyridinium 3-oxide hexafluorophosphate (HATU), HBTU, or HOBt alone in DMF failed to produce any detectable cyclized product [24–26]. Ultimately, successful macrocyclization was achieved by employing benzotriazol-1-yloxytripyrrolidinophosphonium hexafluorophosphate (PyBOP) as the coupling reagent in DMF at a concentration of 10^−3^ M. After cleavage from the resin and global side-chain deprotection, the crude cyclic peptides were purified by preparative RP-HPLC. This optimized protocol afforded segetalins A–H, J and K (**1**–**10**) with 45% to 70% isolated yields (Figure 1).

### Structural characterization

The synthetic compounds **1**–**10** were rigorously characterized to confirm their identity and purity (see Supporting Information File 1). High-resolution electrospray ionization mass spectrometry (HRESIMS) data for all compounds matched the calculated exact masses for their respective molecular formulas. NMR spectroscopic analysis in appropriate deuterated solvents (e.g., DMSO-*d*_6_, D_2_O) fully corroborated the amino acid sequence and cyclic connectivity, demonstrating unequivocal structural identity with the natural isolates. Analytical HPLC confirmed the high purity (>95%) of all synthetic segetalins. However, experimental data for segetalin C revealed the existence of a multi-state conformational equilibrium in solution, which is dependent on solvent polarity.

#### Secondary structure analysis by circular dichroism (CD)

**Figure 2:**
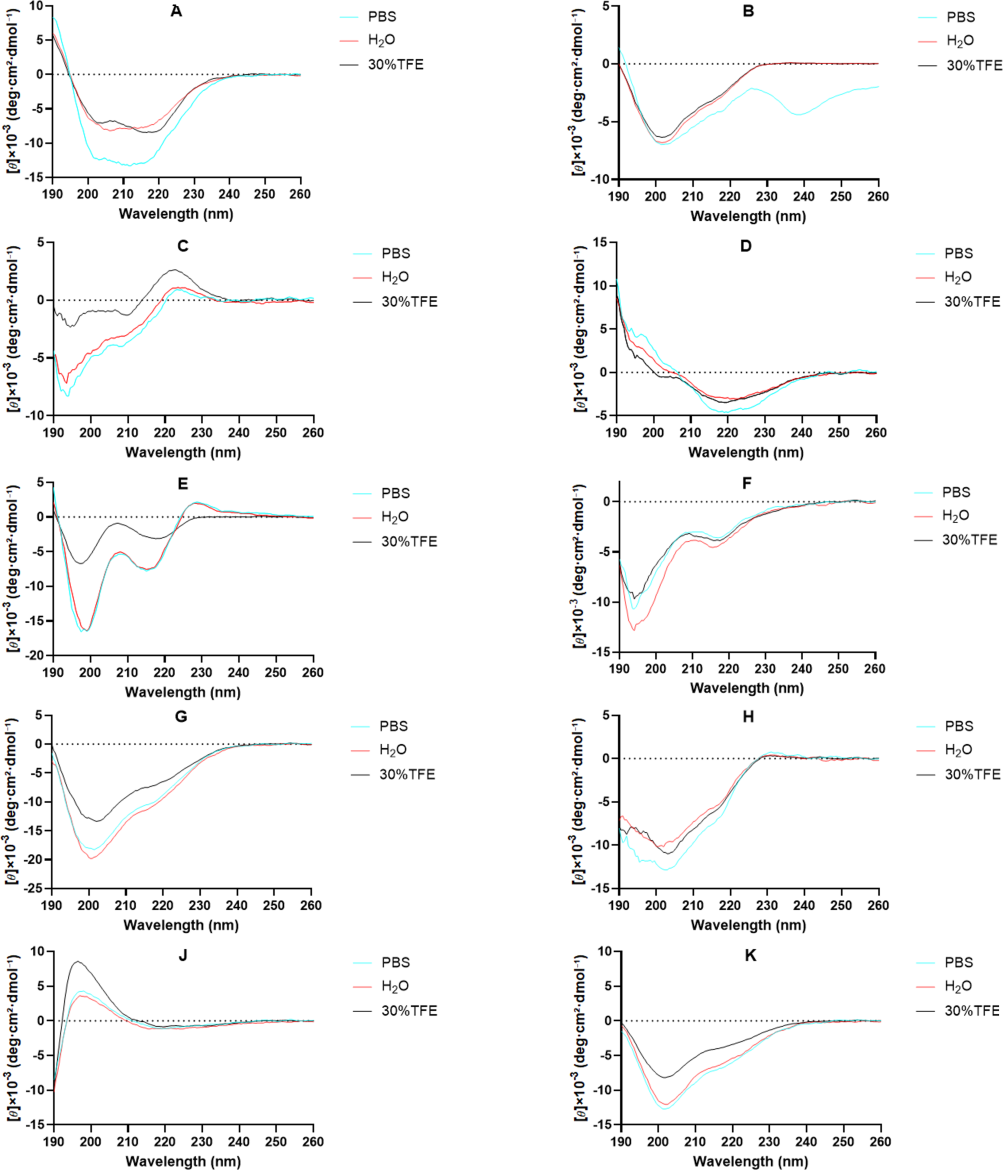
The CD spectra of segetalins A–H, J and K.

The secondary structures of compounds **1**–**10** were investigated using circular dichroism (CD) spectroscopy in aqueous buffer (0.01×PBS), deionized H₂O, and 30% TFE (2,2,2-trifluoroethanol) (Figure 2). CD spectra in the far-UV region (190–250 nm) provide signatures of peptide backbone conformation [27–29]: a random coil typically shows a negative band near 198 nm and a positive band near 218 nm; an α-helix exhibits characteristic minima at 208 nm and 222 nm and a maximum near 192 nm; β-sheet structures are often indicated by a single minimum between 210–220 nm and a maximum below 200 nm.

CD spectroscopy revealed that cyclic topology shifts characteristic peaks and stabilizes secondary structures through cooperative ring size/sequence/solvent effects [30]. TFE disrupts the hydrogen-bonding network of water, reduces solvent polarity, and enhances the stability of intramolecular hydrogen bonds in the peptide backbone, thereby promoting the formation of stable secondary structures (such as α-helices or β-sheets) in cyclic peptides. Spectra acquired in H_2_O, 0.01×PBS, and 30% TFE (Table S1, Supporting Information File 1) demonstrate: (i) definitive β-sheet signatures (217 nm minima) in **1**, **3**, **4**; (ii) enhanced β-sheet stability from constrained macrocycles in **2**, **7**, **8**, **10**; (iii) universal α-helix induction by TFE in **1**–**10**, with **5** and **6** retaining helicity in aqueous buffers.

## Conclusion

We have developed an efficient and reliable solid-phase synthesis strategy for the cyclopeptide family of segetalins A–H, J and K from *Vaccaria segetalis*. Key optimizations include the use of a cost-effective 2-chlorotrityl chloride resin, efficient Fmoc deprotection and linear coupling conditions (HOBt/HBTU), and the identification of PyBOP as a highly effective coupling reagent for the challenging head-to-tail macrocyclization step under moderate dilution (10^−3^ M). This protocol afforded the target cyclic peptides in practical isolated yields (45–70%) and high purity. Comprehensive structural characterization by HRESIMS, NMR, and HPLC confirmed the identity and high purity of the synthetic segetalins. CD spectroscopy provided insights into their secondary structural preferences. This robust and scalable methodology overcomes significant limitations of previous synthetic approaches, providing ample quantities of these bioactive cyclopeptides for detailed biological evaluation and structure–activity relationship studies. The systematic investigation of the their key biological activities, including estrogenic activity (assessed via breast cell proliferation assays), antitumor activity (evaluated through HeLa cell inhibition assays), and antibacterial activity (evaluated against Gram-positive bacteria), will be conducted in our laboratory.

## Supporting Information

File 1 Experimental section, characterization and copies of spectra.

## Data Availability

All data that supports the findings of this study is available in the published article and/or the supporting information of this article.
