# Supplementary material for: Efficient solid-phase synthesis and structural characterization of segetalins A–H, J and K
Source: Beilstein J Org Chem. 2025 Nov 27;21:2612–7. doi: 10.3762/bjoc.21.202 (PMC12667733; doi:10.3762/bjoc.21.202)
Supplement: File 1 — Experimental section, characterization and copies of spectra. [file Beilstein_J_Org_Chem-21-2612-s001.pdf]

BEILSTEIN JOURNAL OF ORGANIC CHEMISTRY

Supporting Information

for

Efficient solid-phase synthesis and structural characterization of segetalins A–H, J and K

Liangyu Liu, Wanqiu Lu, Quanping Guo and Zhaoqing Xu

Beilstein J. Org. Chem. **2025,** *21,* 2612–2617. doi:10.3762/bjoc.21.202

Experimental section, characterization and copies of spectra

License and Terms: This is a supporting information file under the terms of the Creative Commons Attribution License (https://creativecommons.org/licenses/by/4.0). Please note that the reuse, redistribution and reproduction in particular requires that the author(s) and source are credited and that individual graphics may be subject to special legal provisions.
The license is subject to the *Beilstein Journal of Organic Chemistry* terms and conditions: (https://www.beilstein-journals.org/bjoc/terms)

Table of contents

1. General information S2

2. Cyclopeptide synthesis S2

    2.1 General procedure for solid phase peptide synthesis S2

    2.2 General procedure for synthesizing cyclic peptides S4

3. Far UV CD (ultraviolet circular dichroism) assay S4

    3.1 General procedure for CD S4

    3.2 Analysis of the CD spectra revealed distinct structural features. S5

4. Characterization of products S6

5. NMR spectra of products S19

1. General information

All commercially available reagents were used without further purification unless otherwise stated. All solvents were purified and dried according to standard methods prior to use. ^{1}H and ^{13}C NMR spectra were acquired on Bruker AVANCE NEO 600 MHz and 400 MHz liquid-state NMR spectrometers using CD_3OD, DMSO-d_6, D_2O, or CD_3CN as solvents, unless otherwise specified. Data for ^{1}H NMR are recorded as follows: chemical shift (δ, ppm), multiplicity (s = singlet, d = doublet, t = triplet, m = multiplet, q = quartet, dd = doublet of doublets, dt = doublet of triplets, td = triplet of doublets, and brs = broad signal, and coupling constant(s) in Hz integration). Data for ^{13}C NMR is reported in terms of chemical shift (δ, ppm). All new compounds were further characterized by high resolution mass spectra (HRMS, ESI source, Bruker MaXis 4G). Semi-preparative HPLC was performed on Hanbon Sci.& Tech. HPLC analysis was performed on Tongwei EasySep®-3030. CD was performed on a CY-1700 CD spectrometer.

2. Cyclopeptide synthesis

2.1 General procedure for solid-phase peptide synthesis

Solid-phase synthesis of linear peptides (taking the linear segetalin A peptide with the sequence N-Gly-Val-Pro-Val-Trp-Ala-C as an example): synthesized using standard Fmoc solid-phase peptide synthesis (SPPS) technology, all peptide synthesis reactions were carried out at a 4 mmol scale, and the amino acids used were commercially available Fmoc-protected amino acids.

a) Resin swelling: In a dry weighing chamber, weigh out dry dichloro resin (Dichloro Resin, 0.703 mmol/g, 4 mmol scale) and transfer it into a 250 mL synthesis vessel. Add approximately 20 mL of dichloromethane, then agitate for 20–30 minutes to swell the resin. Upon completion of swelling, remove the solvent by vacuum filtration.

b) Loading of the first amino acid: In a dry plastic tube, add Fmoc-ʟ-amino acid (16 mmol, 4 equiv) and dissolve it in approximately 20 mL of dichloromethane. Add DIPEA (24 mmol, 6 equiv), shake to mix uniformly, and transfer the solution into the synthesis vessel. Rinse the plastic tube with dichloromethane and add the rinse solution to the synthesis vessel. Stir the reaction mixture for 1 hour.

c) Capping of unreacted resin sites: Add 5 mL of anhydrous methanol solution, stir for 20 minutes, then remove the solvent by vacuum filtration.

d) Ninhydrin test: Wash the resin with *N*,*N*-dimethylformamide (DMF, 3 × 5 minutes), and aspirate to dryness after completion. Following the second wash, transfer a small amount of resin into a clean test tube, add ninhydrin detection reagent to verify the successful loading of the first amino acid. A successful coupling is indicated by the resin remaining colorless after heating in a water bath for 1–2 minutes.

e) Fmoc deprotection: Add approximately 20 mL of deprotection reagent (1% piperidine + 1% DBU in DMF) to the synthesis vessel and conduct three rounds of deprotection reactions, each followed by solvent aspiration. React for 5 min, 5 min, and 10 min sequentially. Wash the resin with DMF (3 × 5 min), aspirating the solvent after each wash. After the second DMF wash, transfer a small aliquot of resin to a clean test tube and add ninhydrin reagent to confirm successful Fmoc removal. A positive test (blue color development after heating in a water bath for 1–2 min) indicates complete deprotection.

f) Coupling of the second amino acid: In a dry plastic tube, combine Fmoc-L-amino acid (12 mmol, 3 equiv), HBTU (12 mmol, 3 equiv), and HOBt (12 mmol, 3 equiv). Dissolve the mixture in approximately 20 mL of DMF, then add DIPEA (24 mmol, 6 equiv). After thorough mixing, transfer the solution to the synthesis vessel, rinsing the plastic tube with DMF to ensure complete transfer. Stir the reaction mixture for 1 hour, then remove the solvent by vacuum filtration.

g) Coupling of remaining amino acids: Repeat steps d–f sequentially for each subsequent amino acid until the full peptide sequence is assembled. After completing the final coupling step, perform Fmoc deprotection on the *N*-terminus of the last amino acid to expose the free amine group for downstream applications.

h) Resin drying: Sequentially add approximately 20 mL of dichloromethane (2 × 3 minutes) and methanol (2 × 3 minutes) to the synthesis vessel for washing. After completion, aspirate thoroughly until the resin is dried to a powdery state.

i) Peptide cleavage: Add approximately 50 mL of cleavage cocktail (1% TFA in DCM) to the synthesis vessel and stir for 1.5 hours. Collect the filtrate containing the cleaved peptide. Repeat the cleavage process twice more using fresh cleavage cocktail (2 × 10 minutes), combining all filtrates. The reaction mixture was concentrated, and precipitated out by the addition of cold ethyl ether to obtain the crude product.

2.2 General procedure for synthesizing cyclic peptides

a) Liquid-phase cyclization: In a dry 500 mL round-bottomed flask equipped with a magnetic stirring bar, combine the crude linear peptide from the previous step (0.3 mmol, 1 equiv) and PyBOP (0.6 mmol, 2 equiv). Dissolve the solids in 300 mL of anhydrous DMF. With vigorous stirring, slowly add DIPEA (0.9 mmol, 3 equiv) dropwise via syringe. Allow the reaction to proceed at room temperature overnight under nitrogen atmosphere. Following completion, remove DMF under reduced pressure using a rotary evaporator.

b) Side-chain deprotection: Add approximately 50 mL of cleavage cocktail (TFA/DCM 1:1) or TFA/TIS/H_2O 95:2.5:2.5) directly to the round-bottomed flask containing the cyclized peptide. Stir the reaction mixture at room temperature for 1.5 hours to remove all acid-labile side-chain protecting groups. Following deprotection, concentrate the mixture under reduced pressure using a rotary evaporator. Precipitate the crude peptide by slowly adding ice-cold diethyl ether. Collect the precipitate by filtration or centrifugation to obtain the crude cyclic peptide product.

c) The crude product was purified by semi-HPLC using acetonitrile/H_2O as the gradient elution (Hanbon Sci.& Tech (Dubhe, C18, 10 µ, 100 Å), length 20 mm × 250 mm, 5–75% CH_3CN/H_2O + 0.1% trifluoroacetic acid). The purity of all peptides was determined by HPLC analysis (Waters e2695 (BEH, C18, 10 µ, 130 Å), length 4.6 mm × 250 mm, 5–95% CH_3CN/H_2O + 0.1% trifluoroacetic acid). The pure peptides were obtained as a compound after lyophilization.

3. Far UV CD (ultraviolet circular dichroism) assay

3.1 General procedure for CD

A JASCO J-1500 CD Spectropolarimeter was employed to perform Far UV CD analysis at 25 °C. Samples of 0.1–0.3 mg/mL were reconstituted in aqueous buffer (0.01 × PBS), deionized H_2O, and 30% TFE. Far UV CD spectra were collected from 195 nm to 260 nm on a 0.1 cm path length cuvette at a data pitch of 0.5 nm, bandwidth of 1.0 nm, and a scan speed of 50 nm/min. PBS buffer was used to perform blank correction. Equation $[\theta] = (M \times 0.001 \times \theta)/ (c \times N)$, where c is the sample concentration (mg/mL), N is the number of amino acids, θ is the measured ellipticity (mdeg) was used to calculate molar ellipticity.

3.2 Analysis of the CD spectra revealed distinct structural features

Table S1: The CD secondary structure and features of **1**–**10**.

Segetalins	Solvent	Secondary structure tendency (preliminary inference)	Key CD features
1	0.01×PBS	β-turn/β-sheet (partial α-helix fragments)	positive peak at ≈200 nm + negative peak at ≈220 nm; weak double minima at 208/222 nm
	H_2O	β-turn/β-sheet (weakened, random coil increased)	broadened peaks at 200/220 nm; reduced peak intensity
	30% TFE	α-helix enhanced (clear double minima)	sharp double negative peaks at 208/222 nm
2	0.01×PBS	β-sheet (pronounced)/random coil (flat spectra)	single negative peak at ≈217 nm; flat baseline → random coil
	H_2O	β-sheet (broadened)/random coil (reduced intensity)	217 nm peak widened; intensity decreased by ≈30% vs PBS
	30% TFE	α-helix (induced)/β-sheet (strengthened)	new double minima at 208/222 nm; enhanced 217 nm peak (if β-sheet dominant)
3	PBS	α-helix (weak)/β-sheet (possible)	faint double minima at 208/222 nm; 217 nm shoulder
	H_2O	random coil (increased)/α-helix (diminished)	reduced 208/222 nm signals; flattened baseline
	30% TFE	α-helix (strongly enhanced)	intense double negative peaks at 208/222 nm
4	0.01×PBS	β-sheet (dominant)	strong 217 nm negative peak
	H_2O	β-sheet (weakened)/random coil	217 nm peak intensity reduced by 40%
	30% TFE	β-sheet (strengthened)	217 nm peak
5	0.01×PBS	α-helix + β-sheet (mixed)	double minima at 208/222 nm + 217 nm peak
	H_2O	random coil (major)/residual structures	weakened peaks; no dominant features
	30% TFE	α-helix (predominant)	prominent 208/222 nm double minima
6	0.01×PBS	α-helix (moderate)	clear double minima at 208/222 nm
	H_2O	α-helix (weakened)/random coil	reduced intensity of 208/222 nm peaks
	30% TFE	α-helix (highly stable)	sharp, intense double negative peaks at 208/222 nm

7	0.01×PBS	β-sheet (moderate)/random coil (weak signals)	217 nm negative peak (medium intensity); faint features → random coil
	H_2O	random coil (dominant)/residual β-sheet	217 nm peak flattened; mostly flat baseline
	30% TFE	β-sheet (markedly strengthened)	intense negative peak at 217 nm
8	0.01×PBS	random coil (main)/β-sheet (trace)	flat spectra; weak 217 nm shoulder
	H_2O	random coil (exclusive)	no distinct peaks; smooth baseline
	30% TFE	α-helix (weakly induced)	shallow double minima at 208/222 nm
9	0.01×PBS	β-sheet/α-helix (minor)	217 nm peak + faint 208/222 nm minima
	H_2O	random coil (significant)/β-sheet (residual)	217 nm peak broadened; low intensity
	30% TFE	α-helix (induced)	new double minima at 208/222 nm (strong intensity)
10	0.01×PBS	β-sheet (strong)	sharp negative peak at 217 nm
	H_2O	β-sheet (retained)/random coil (minor)	217 nm peak preserved (slight broadening)
	30% TFE	α-helix + β-sheet (mixed)	double minima at 208/222 nm + 217 nm negative peak

4. Characterization of products

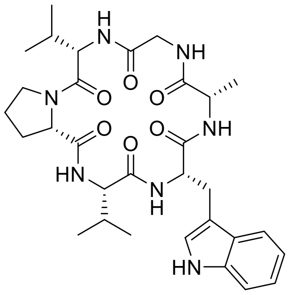

Segetalin A

***Cyclo*-[Gly-Val-Pro-Val-Trp-Ala] (segetalin A):**

^{1}H NMR (400 MHz, DMSO-d_6) δ 10.91 (s, 1H), 9.00 (d, J = 6.8 Hz, 1H), 7.55 (d, J = 8.0 Hz, 1H), 7.47 (t, J = 5.2 Hz, 1H), 7.42 (d, J = 9.2 Hz, 1H), 7.33 (t, J = 8.0 Hz, 2H), 7.15 (d, J = 2.0 Hz, 1H), 7.08 (q, J = 6.8 Hz, 1H), 6.98 (t, J = 7.2 Hz, 1H), 4.52 (q, J = 4.8 Hz, 1H), 4.35 (d, J = 8.0 Hz, 1H), 4.27 (q, J = 6.4 Hz, 1H), 4.14 (t, J = 10.0 Hz, 1H), 3.72-3.56 (m, 2H), 3.58 (q, J = 4.0 Hz, 1H), 3.35

(dd, J = 5.6, 16.4 Hz, 1H), 3.18-3.06 (m, 2H), 1.95-1.88 (m, 2H), 1.69-1.65 (m, 1H), 1.15 (d, J = 7.2 Hz, 3H), 0.96 (d, J = 6.8 Hz, 3H), 0.83 (t, J = 6.8 Hz, 3H), 0.77 (d, J = 4.4 Hz, 3H), 0.76 (d, J = 4.4 Hz, 3H).

^{13}C NMR (100 MHz, DMSO-d_6) δ 173.60, 173.29, 172.05, 171.61, 171.33, 169.72, 136.58, 127.55, 123.92, 121.62, 118.99, 118.87, 111.84, 109.67, 60.82, 60.41, 56.02, 55.62, 49.98, 47.41, 43.33, 31. 81, 30.46, 30.16, 26.50, 21.96, 20.07, 19.70, 19.14, 17.81, 15.94.

HRMS (ESI) $C_{31}H_{43}N_7O_6$ $[M+H]^+$ calcd:610.3348, found:610.3290.

Analytical HPLC of segetalin A

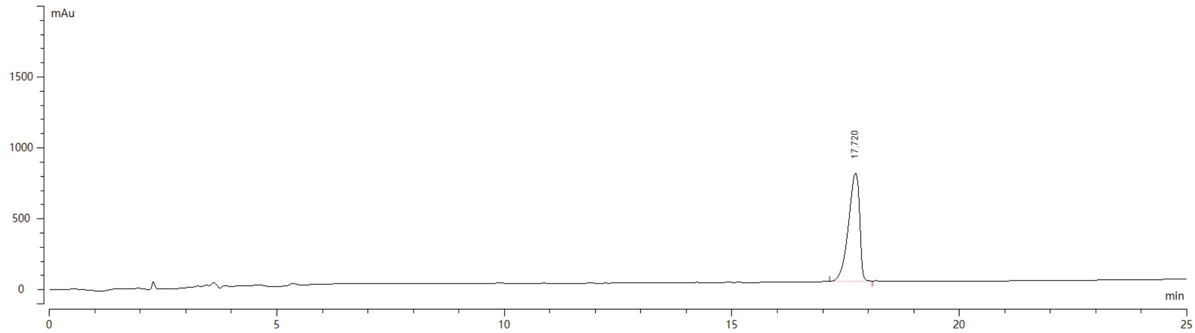

Compound	Ret. time (min)	Area (mAu*s)	Area (%)	Height (mAU)
Segetalin A	17.720	12913.889	100.000	762.374

High-resolution MS of segetalin A

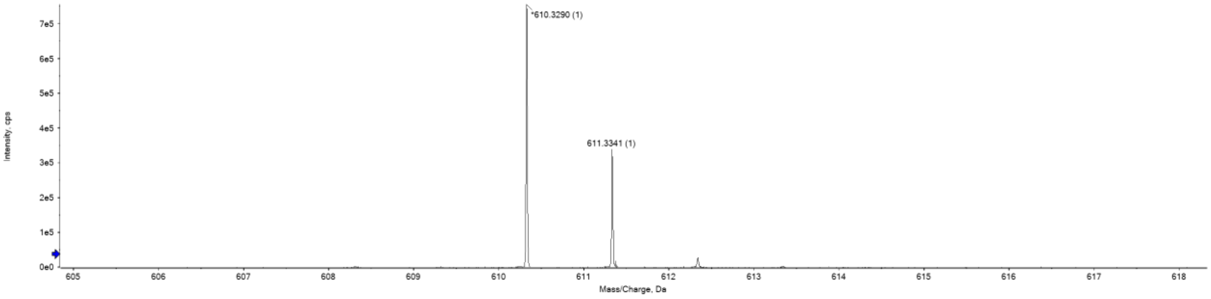

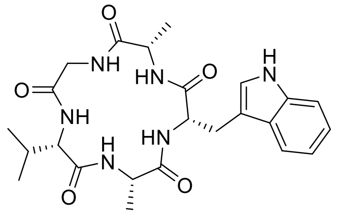

Segetalin B

***Cyclo*-[Gly-Val-Ala-Trp-Ala] (segetalin B)**

^{1}H NMR (400 MHz, DMSO-d_6) δ 10.93 (d, *J* = 1.2 Hz, 1H), 8.50 (t, *J* = 5.6 Hz, 1H), 8.06-8.00 (m, 3H), 7.82 (d, *J* = 8.4 Hz, 1H), 7.61 (d, *J* = 8.0 Hz, 1H), 7.39 (d, *J* = 8.0 Hz, 1H), 7.18 (d, *J* = 1.6 Hz, 2H), 7.12 (t, *J* = 7.2 Hz, 1H), 7.04 (t, *J* = 7.2 Hz, 1H), 4.30-4.26 (m, 2H), 4.19-4.11 (m, 2H), 3.97 (t, *J* = 7.2 Hz, 1H), 3.37 (dd, *J* = 5.2 Hz, 14.4 Hz, 1H), 3.32-3.21 (m, 2H), 2.07-1.99 (m, 1H), 1.28 (dd, *J* = 6.8 Hz, 11.6 Hz, 6H), 0.93 (t, *J* = 6 Hz, 6H).

^{13}C NMR (100 MHz, DMSO-d_6) δ 172.12, 171.95, 171.60, 170.86, 170.22, 127.78, 124.12, 121.53, 118.88, 118.85, 111.96, 110.53, 60.01, 56.74, 50.19, 48.96, 43.94, 30.20, 26.92, 19.70, 18.57, 17.72, 17.60.

HRMS (ESI) $C_{24}H_{32}N_6O_5$ $[M+Na]^+$ calcd:507.2337, found:507.2326.

Analytical HPLC of segetalin B

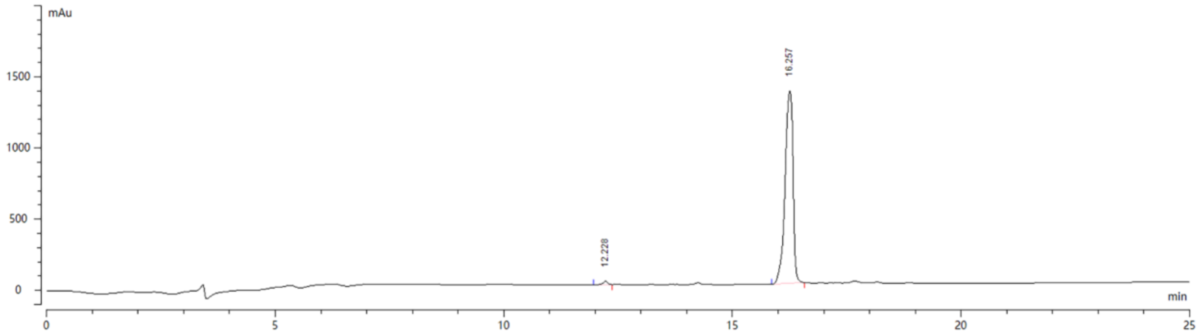

Compound	Ret. time (min)	Area (mAu*s)	Area (%)	Height (mAU)
N.A.	12.228	165.220	1.000	23.786
Segetalin B	16.257	16354.437	99.000	1352.468

High-resolution MS of segetalin B

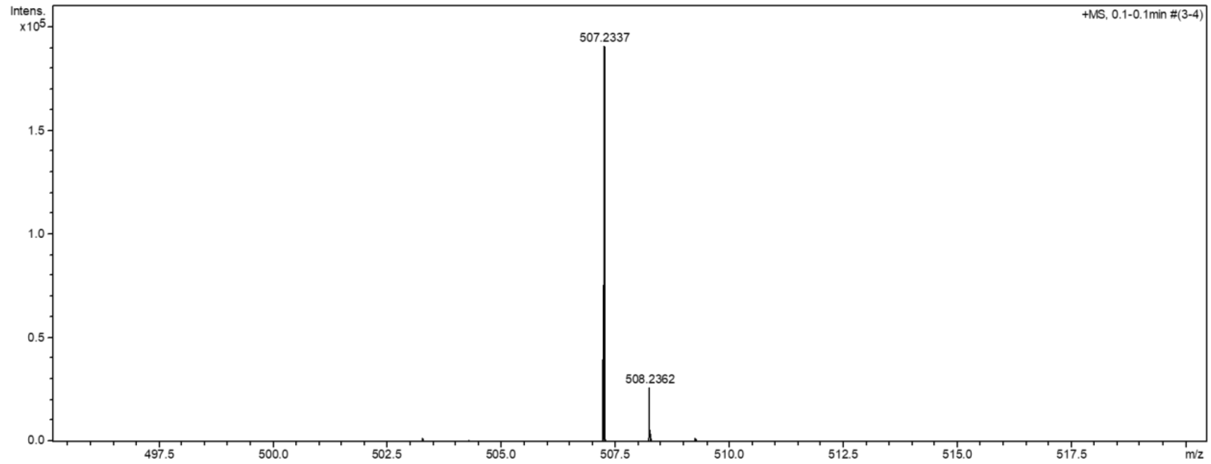

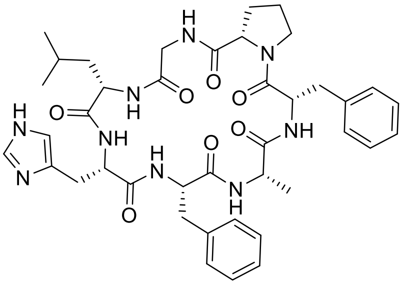

Segetalin C

***Cyclo*-[Gly-Leu-His-Phe-Ala-Phe-Pro] (segetalin C)**

^{1}H NMR (400 MHz, Pyridine-*d*$_5$) δ 10.26 (q, *J* = 3.8 Hz, 1H), 10.13 (d, *J* = 9.2 Hz, 1H), 9.42 (d, *J* = 3.8 Hz, 1H), 8.74 (d, *J* = 10.4 Hz, 1H), 8.65 (d, *J* = 5.3 Hz, 1H), 8.15 (s, 1H), 8.03 (d, *J* = 7.7 Hz, 1H), 7.56 (s, 1H), 7.51 (d, *J* = 7.3 Hz, 2H), 7.41-7.36 (m, 4H), 7.31 (t, *J* = 7.2 Hz, 2H), 7.26-7.22 (m, 2H), 7.20 (s, 1H), 5.41-5.29 (m, 1H), 5.13-5.07 (m, 1H), 5.00-4.88 (m, 3H), 4.40 (t, *J* = 7.8 Hz, 1H), 3.95 (dd, *J* = 15.1 Hz, 6.0 Hz, 1H), 3.86 (dd, *J* = 16.9 Hz, 3.8 Hz, 1H), 3.75 (dd, *J* = 15.0 Hz, 7.8 Hz, 1H), 3.57 (dd, *J* = 14.6 Hz, 4.8 Hz, 1H), 3.37 (dd, *J* = 14.5 Hz, 9.0 Hz, 1H), 3.22-3.16 (m, 1H), 3.13 (dd, *J* = 12.8 Hz, 3.5 Hz, 1H), 3.00-2.94 (m, 1H), 2.67 (q, *J* = 8.5 Hz, 1H), 1.96-1.81 (m, 5H), 1.79 (d, *J* = 7.4 Hz, 3H), 1.53-1.48 (m, 1H), 1.29-1.22 (m, 1H), 0.84 (d, *J* = 6.4 Hz, 3H), 0.76 (d, *J* = 6.4 Hz, 3H).

^{13}C NMR (100 MHz, pyridine-*d*$_5$) 174.53, 174.13, 173.59, 173.39, 173.05, 171.26, 171.17, 139.28, 138.87, 137.55, 136.42, 131.39, 130.78, 130.20, 128.46, 128.38, 118.33, 63.47, 59.89, 55.47, 55.13, 50.75, 49.31, 45.15, 45.07, 40.34, 38.43, 30.89, 30.22, 26.18, 26.08, 24.12, 23.20, 19.57.

HRMS (ESI) $C_{40}H_{51}N_9O_7$ $[M+H]^+$ calcd:770.3985, found:770.3904.

Analytical HPLC of segetalin C

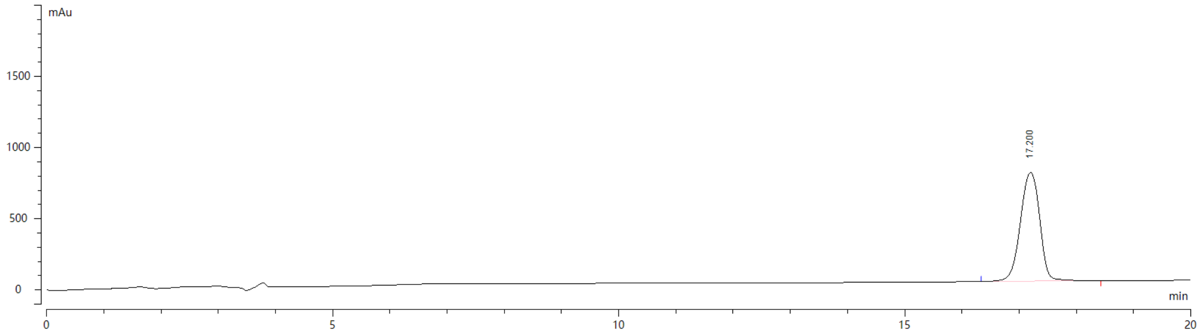

Compound	Ret. time (min)	Area (mAu*s)	Area (%)	Height (mAU)
Segetalin C	17.200	17899.057	100.000	763.047

High-resolution MS of segetalin C

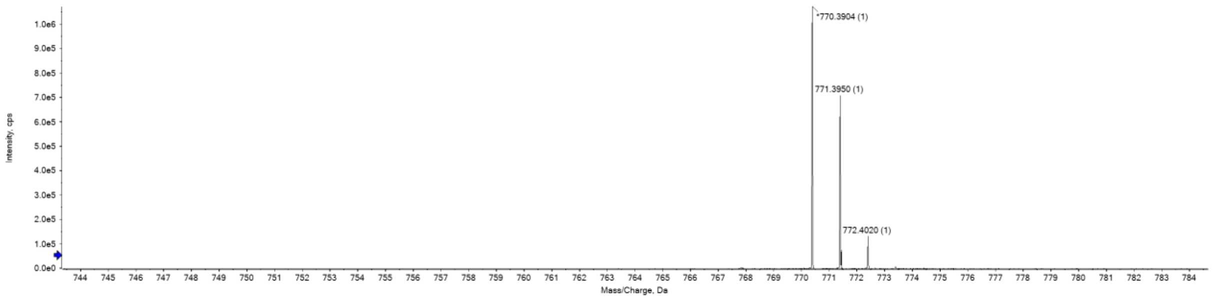

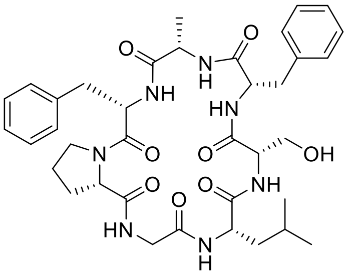

Segetalin D

***Cyclo*-[Gly-Leu-Ser-Phe-Ala-Phe-Pro] (segetalin D)**

^{1}H NMR (600 MHz,CD_3CN) δ 8.30 (d, *J* = 6.0 Hz, 1H), 8.04 (d, *J* = 10.2 Hz, 1H), 7.44 (s, 1H), 7.32-7.20 (m, 1H), 7.16 (s, 1H), 6.97 (d, *J* = 8.4 Hz, 1H), 4.82 (q, *J* = 8.4 Hz, 1H), 4.60-4.58 (m, 1H), 4.50-4.49 (m, 1H), 4.34-4.22 (m, 3H), 4.09 (t, *J* = 7.2 Hz, 1H), 4.05 (dd, *J* = 2.4 Hz, 11.4Hz, 1H), 3.92 (d, *J* = 10.8 Hz, 1H), 3.50 (t, *J* = 10.2 Hz, 1H), 3.37 (dd, *J* = 3.6 Hz, 16.8 Hz, 1H), 3.10 (dd, *J* = 4.8 Hz, 14.4 Hz, 1H), 3.06-3.31 (m, 2H), 2.93-2.89 (m, 1H), 2.74 (dd, *J* = 6.6 Hz, 13.2 Hz, 1H), 2.13-2.10 (m, 1H), 1.87-1.85 (m, 1H), 1.82-1.77 (m, 2H), 1.68-1.58 (m, 3H), 1.11 (d, *J* = 7.1 Hz, 3H), 0.85 (d, *J* = 6.0 Hz, 3H), 0.80 (d, *J* = 6.0 Hz, 3H).

^{13}C NMR (150 MHz, CD_3CN) δ 171.60, 171.49, 171.19, 170.43, 170.34, 169.46, 168.80, 136.61, 136.49, 129.21, 128.77, 128.20, 128.01, 126.58, 126.32, 62.23, 61.60, 56.77, 54.13, 52.62, 52.00, 48.97, 47.50, 42.42, 38.68, 35.68, 28.88, 24.23, 24.10, 21.82, 20.67, 17.34. **HRMS (ESI)** $C_{37}H_{49}N_7O_8$ $[M+H]^+$ calcd:720.3716, found:720.3659.

Analytical HPLC of segetalin D

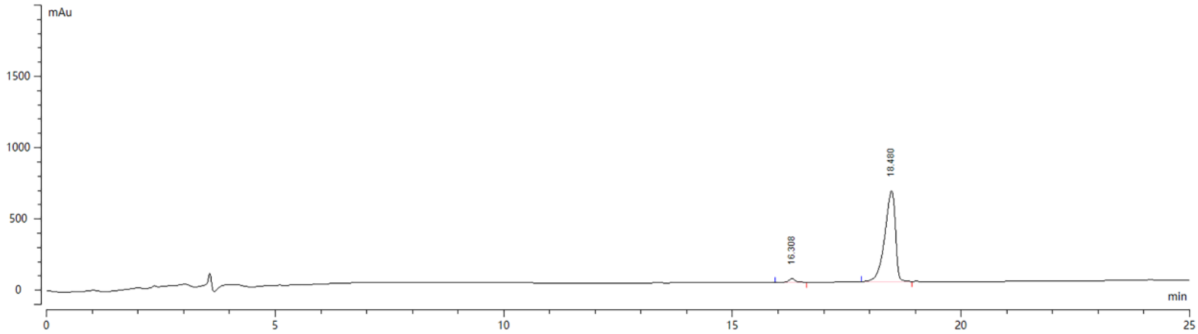

Compound	Ret. time (min)	Area (mAu*s)	Area (%)	Height (mAU)
N.A.	16.308	253.379	2.423	25.741
Segetalin D	18.480	10203.303	97.577	635.409

High-resolution MS of segetalin D

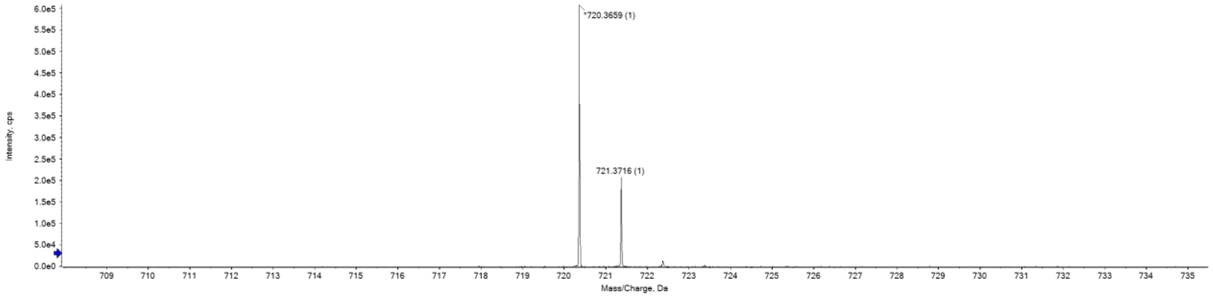

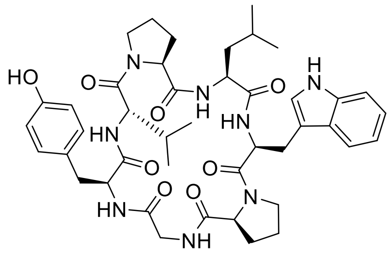

Segetalin E

***Cyclo*-[Gly-Tyr-Val-Pro-Leu-Trp-Pro] (segetalin E)**

^1H NMR (600 MHz,CD_3OD) δ 8.41 (t, J = 4.8 Hz, 1H), 7.79 (d, J = 6.0 Hz, 1H), 7.68 (d, J = 7.8 Hz, 1H), 7.45 (d, J = 7.8 Hz, 1H), 7.35 (d, J = 7.8 Hz, 1H), 7.27-7.26 (m, 2H), 7.13-7.10 (m, 1H), 7.08-7.05 (m, 1H), 7.03-7.01 (m, 1H), 6.98 (d, J = 7.8 Hz, 1H), 6.73 (d, J = 8.4 Hz, 1H), 6.62 (d, J = 8.4

Hz, 1H), 5.04 (t, J = 5.2 Hz, 1H), 4.60-4.58 (m, 2H), 4.22-4.15 (m, 2H), 3.95 (dd, J = 4.0 Hz, 7.0Hz, 1H), 3.80 (t, J = 8.1 Hz, 1H), 3.68 (q, J = 16.8 Hz, 2H), 3.60-3.45 (m, 3H), 3.37 (t, J = 10.4 Hz, 1H), 3.25 (dd, J = 5.3 Hz, 14.9 Hz, 1H), 2.84-2.80 (m, 1H), 2.66 (dd, J = 3.5 Hz, 13.7 Hz, 1H), 2.46-2.43 (m, 1H), 2.28-2.25 (m, 1H), 2.12-2.11 (m, 1H), 2.00-1.88 (m, 4H), 1.73-1.68 (m, 1H), 1.62-1.57 (m, 2H), 1.52-1.47 (m, 1H), 1.36-1.32 (m, 1H), 1.03 (d, J = 6.8 Hz, 3H), 0.96 (d, J = 6.9 Hz, 3H), 0.86 (d, J = 6.5 Hz, 3H), 0.81 (d, J = 2.8 Hz, 3H). **^{13}C NMR (150 MHz, CD_3OD)** δ 173.13, 172.98, 171.87, 171.66, 171.48, 171.31, 169.45, 155.27, 136.11, 129.93, 128.74, 127.94, 124.35, 121.00,118.60, 117.90, 114.44, 111.07, 107.91, 62.53, 61.01, 58.73, 55.01, 54.53, 52.61, 46.02, 42.10, 39.83, 35.34, 30.56, 29.49, 28.51, 26.18, 25.34, 24.92, 21.94, 21.22, 19.81, 18.04, 17.54.

HRMS (ESI) $C_{43}H_{56}N_8O_8$ $[M+H]^+$ calcd:813.4294, found:813.4247.

Analytical HPLC of segetalin E

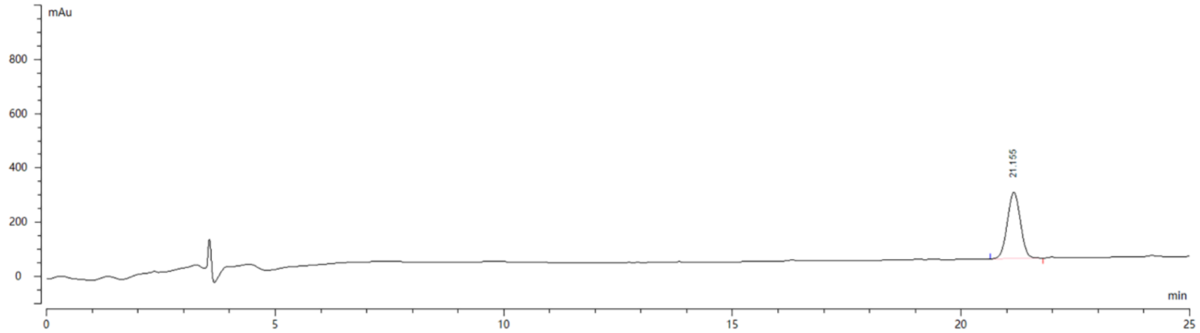

Compound	Ret. time (min)	Area (mAu*s)	Area (%)	Height (mAU)
Segetalin E	21.155	4865.282	100.000	244.294

High-resolution MS of segetalin E

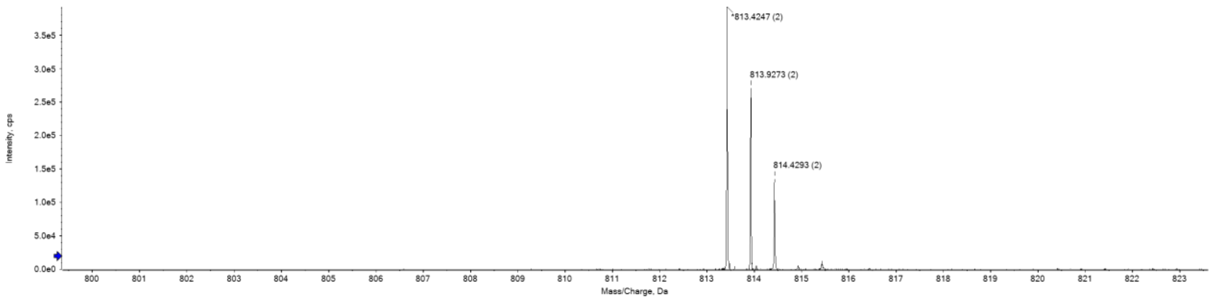

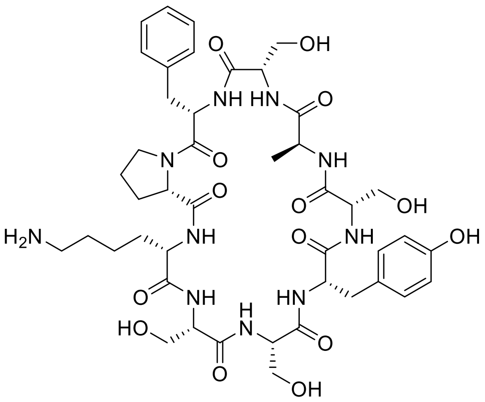

Segetalin F

***Cyclo*-[Tyr-Ser-Ser-Lys-Pro-Phe-Ser-Ala-Ser] (segetalin F)**

^{1}H NMR (400 MHz, D_2O) δ 7.19 (d, *J* = 7.2 Hz, 2H), 7.16 (d, *J* = 6.8 Hz, 1H), 7.10-7.08 (m, 2H), 6.95 (d, *J* = 8.6 Hz, 2H), 6.65 (d, *J* = 8.5 Hz, 1H), 4.50 (t, *J* = 12.2 Hz, 1H), 4.45-4.42 (m, 1H), 4.35-4.28 (m, 2H), 4.25-4.21 (m, 3H), 4.19-4.15 (m, 1H), 4.12 (q, *J* = 7.3 Hz, 1H), 3.75-3.70 (m, 2H), 3.67-3.56 (m, 2H), 3.28-3.16 (m, 2H), 2.97-2.80 (m, 7H), 2.32-2.22 (m, 1H), 1.91-1.82 (m, 3H), 1.64-1.53 (m, 1H), 1.54-1.48 (m, 2H), 1.31-1.25 (m, 2H), 1.21 (d, *J* = 7.2 Hz, 3H).

^{13}C NMR (100 MHz, D_2O) δ 175.40, 174.84, 172.96, 172.64, 171.45, 171.36, 171.32, 170.95, 169.38, 154.44, 135.92, 130.51, 129.07, 128.74, 127.75, 127.24, 115.41, 61.17, 60.95, 60.87, 59.45, 55.54, 55.40, 55.21, 55.16, 54.90, 52.63, 49.84, 49.73, 46.49, 39.16, 37.12, 36.97, 36.07, 35.79, 30.13, 29.99, 29.73, 26.31, 26.09, 23.66, 21.92, 21.83, 16.36.

HRMS (ESI) $C_{35}H_{53}N_9O_{13}$ $[M+H]^+$ calcd:955.4513, found:955.4520.

Analytical HPLC of segetalin F

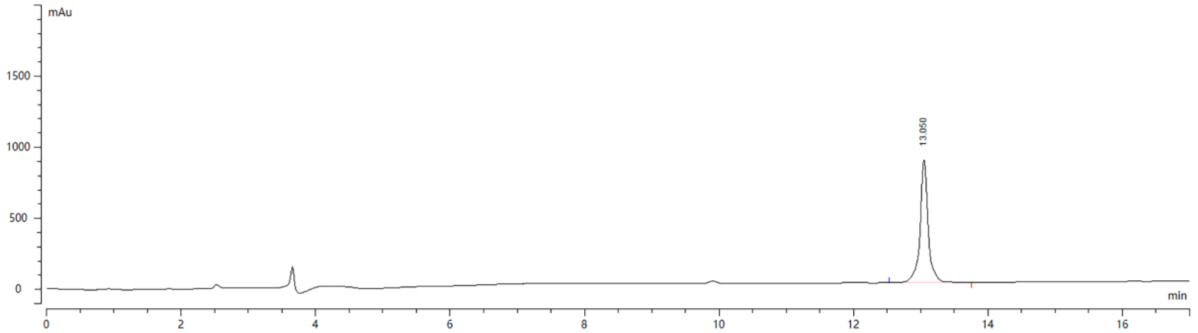

Compound	Ret. time (min)	Area (mAu*s)	Area (%)	Height (mAU)
Segetalin F	8.677	5118.598	99.753	629.009
N.A.	8.938	12.663	0.247	2.532

High-resolution MS of segetalin F

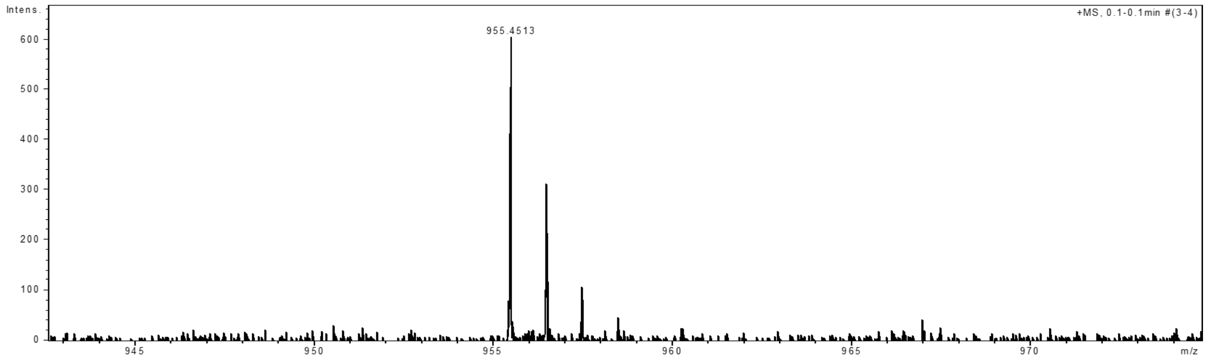

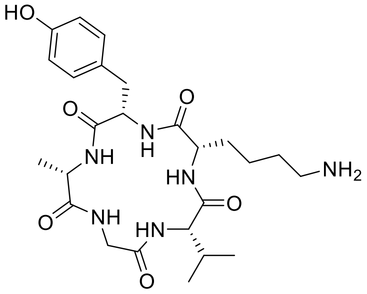

Segetalin G

Cyclo-[Gly-Val-Lys-Tyr-Ala] (segetalin G)

^{1}H NMR (600 MHz, D_2O) δ 8.29 (t, *J* = 5.1 Hz, 1H), 8.17 (dd, *J* = 8 Hz, 15.9 Hz, 2H), 7.93 (d, J = 8.6 Hz, 1H), 7.82 (s, 4H), 7.04 (d, *J* = 8.0 Hz, 2H), 6.73 (d, *J* = 7.9 Hz, 2H), 4.30(q, *J* = 7.6 Hz, 2H), 4.13-4.10 (m, 2H), 3.98 (t, *J* = 8.0 Hz, 1H), 3.47 (dd, *J* = 4.8 Hz, 14.3 Hz, 1H), 3.00 (dd, *J* = 5.1 Hz, 13.5Hz, 1H), 2.93-2.89 (m, 1H), 2.77-2.75 (m, 2H), 2.05-2.01 (m, 1H), 1.62-1.53 (m, 4H), 1.28(d, *J* = 6.8 Hz, 3H), 1.19-1.17 (m, 1H), 1.04 (m, 1H), 0.93(t, *J* = 7.7 Hz, 6H). **^{13}C NMR (150 MHz, D_2O)** δ 172.32, 171.20, 171.09, 170.97, 169.99, 156.54, 130.59, 128.10, 115.59, 60.87, 56.80, 54.68, 49.06, 43.66, 39.21, 36.63, 31.78, 30.24, 27.17, 22.90, 19.80, 18.95, 17.28.

HRMS (ESI) $C_{25}H_{38}N_6O_6$ $[M+H]^+$ calcd:519.2926, found:519.2861.

Analytical HPLC of segetalin G

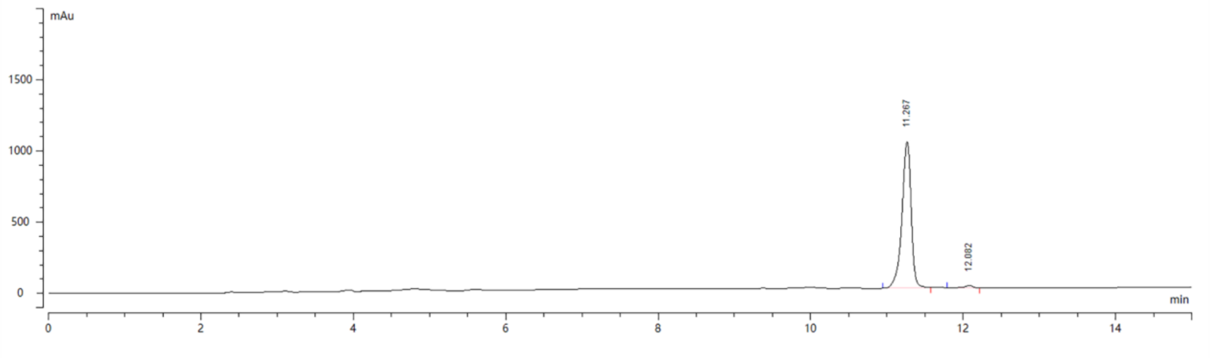

Compound	Ret. time (min)	Area (mAu*s)	Area (%)	Height (mAU)
Segetalin G	13.050	7242.303	100.000	861.399

High-resolution MS of segetalin G

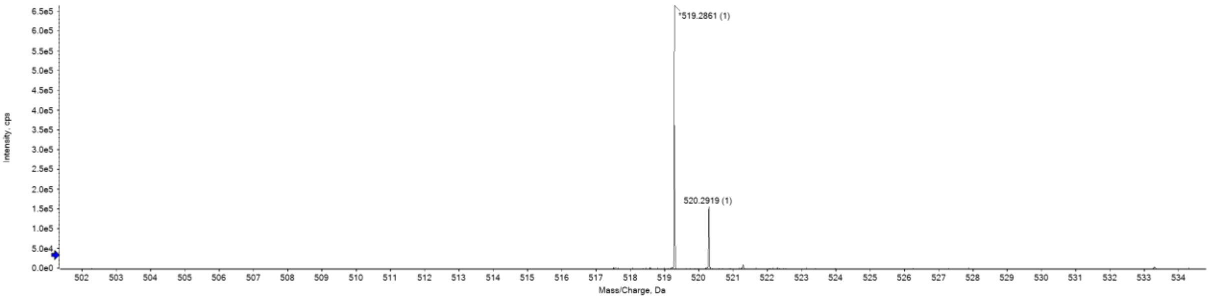

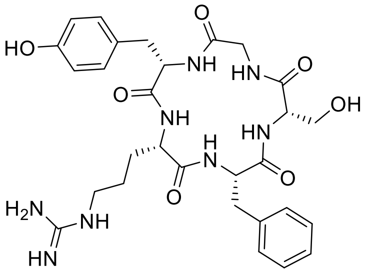

Segetalin H

***Cyclo*-[Gly-Tyr-Arg-Phe-Ser] (segetalin H)**

^{1}H NMR (600 MHz, D_2O) δ 8.45 (t, *J* = 5.14Hz, 1H), 8.36 (d, *J* = 8.1 Hz, 1H), 8.13 (d, *J* = 8.2 Hz, 1H), 8.05 (d, *J* = 8.2 Hz, 1H), 7.84 (d, *J* = 8.3 Hz, 1H), 7.69 (s, 1H), 7.28 (t, *J* = 7.7 Hz, 2H), 7.22-7.19 (m, 3H), 7.02(d, *J* = 8.3 Hz, 3H), 6.67 (d, *J* = 8.3 Hz, 2H), 4.28-4.17 (m, 3H), 4.04 (q, *J* = 7.8 Hz, 1H), 3.85 (dd, *J* = 6.2 Hz, 14.5Hz, 1H), 3.68-3.65 (m, 1H), 3.61-3.58 (m, 1H), 3.43 (dd, *J* = 5.3 Hz, 14.0 Hz, 1H), 3.09 (d, *J* = 7.9 Hz, 2H), 3.06-3.01 (m, 2H), 2.93 (dd, *J* = 3.3 Hz, 14.0 Hz, 1H), 2.74 (dd, *J* = 9.8 Hz, 13.7 Hz, 1H), 1.57 (dd, *J* = 7.5 Hz, 15.0Hz, 2H), 1.31-1.27 (m, 1H), 1.17-1.12 (m, 1H).

^{13}C NMR (150 MHz, D_2O) δ 171.55, 171.48, 171.44, 171.17, 169.71, 157.36, 156.51, 138.12, 130.38, 129.65, 128.84, 128.14, 127.04, 115.67, 61.31, 57.66, 56.74, 55.74, 54.33, 43.71, 40.87, 36.90, 36.75, 29.12, 25.56.

HRMS (ESI) $C_{29}H_{38}N_8O_7$ $[M+H]^+$ calcd:611.2937, found:611.2848.

Analytical HPLC of segetalin H

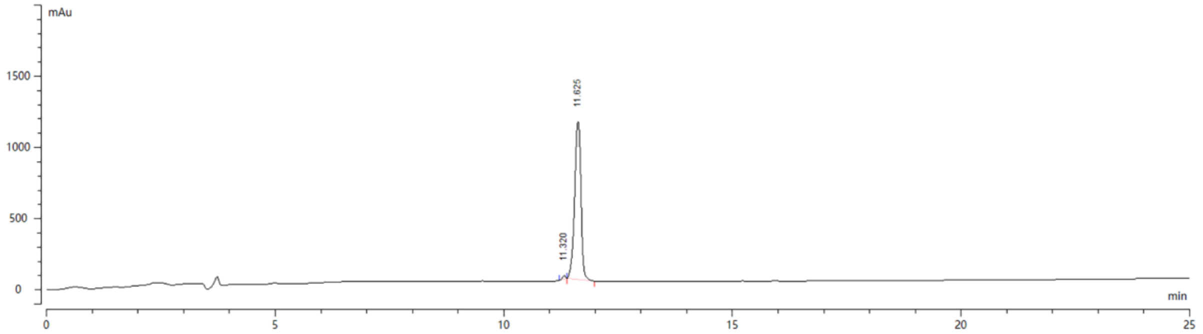

Compound	Ret. time (min)	Area (mAu*s)	Area (%)	Height (mAU)
N.A.	11.320	109.096	1.007	23.984
SegetalinH	11.625	10727.998	98.993	1109.375

High-resolution MS of segetalin H

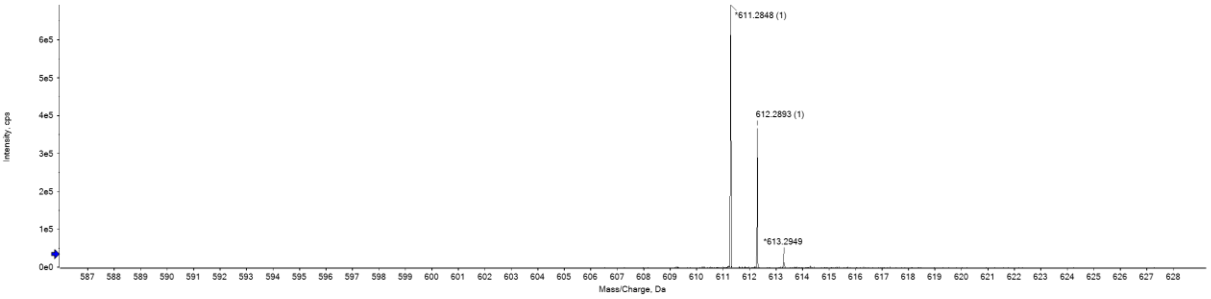

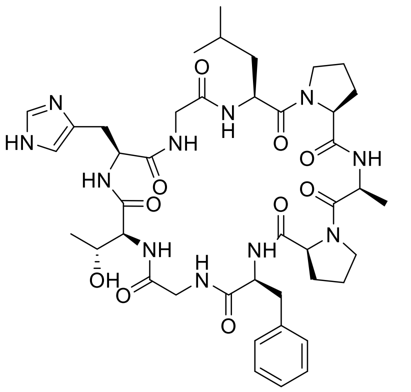

Segetalin J

***Cyclo*-[Phe-Gly-Thr-His-Gly-Leu-Pro-Ala-Pro] (segetalin J)**

^{1}H NMR (600 MHz,CD_3OD) δ 8.81 (d, J = 3.0 Hz, 1H), 8.63 (d, J = 12.0 Hz, 1H), 8.59 (d, J = 3.0 Hz, 1H), 8.47 (d, J = 6.0 Hz, 1H), 8.03 (t, J = 7.8 Hz, 1H), 7.68 (s, 1H), 7.45-7.43 (m, 2H), 7.32-

7.22 (m, 6H), 7.21-7.18 (m, 1H), 4.75-4.72 (m, 1H), 4.67-4.63 (m, 1H), 4.49-4.45 (m, 2H), 4.43 (d, *J* = 12.0 Hz, 1H), 4.34 (dd, *J* = 4.2 Hz, 9.6Hz, 1H), 4.26-4.25 (m, 1H), 4.20-4.16 (m, 1H), 4.14 (dd, *J* = 3.0 Hz, 6.0Hz, 1H), 4.12-4.08 (m, 1H), 3.84-3.80 (m, 2H), 3.66-3.62 (m, 1H), 3.58 (dd, *J* = 4.2 Hz, 15.6Hz, 1H), 3.54-3.50 (m, 1H), 3.34-3.33 (m, 1H), 3.27 (dd, *J* = 3.6 Hz, 13.8 Hz, 1H), 3.21-3.17 (m, 2H), 2.36-2.31 (m, 1H), 2.22-2.16 (m, 2H), 2.13-2.02 (m, 3H), 1.98-1.91 (m, 2H), 1.79-1.74 (m, 1H), 1.61-1.55 (m, 2H), 1.33 (d, *J* = 7.2 Hz, 3H), 1.20 (d, *J* = 6.0 Hz, 3H), 0.92 (d, *J* = 6.6 Hz, 3H), 0.79 (d, *J* = 6.6 Hz, 3H).

^{13}C NMR (150 MHz, CD_3OD) δ 173.85, 173.78, 173.39, 172.17, 171.59, 171.40, 171.00, 170.50, 170.17, 136.94, 133.45, 130.44, 128.57, 128.27, 126.64, 117.01, 67.03, 60.62, 59.41, 58.38, 56.17, 53.29, 49.53, 48.75, 46.49, 44.15, 42.57, 40.15, 36.56, 30.53, 29.43, 24.83, 24.58, 24.42, 22.30, 20.90, 19.71, 18.37, 14.25.

HRMS (ESI) $C_{42}H_{59}N_{11}O_{10}$ $[M+H]^+$ calcd:878.4520, found:878.4408.

Analytical HPLC of segetalin J

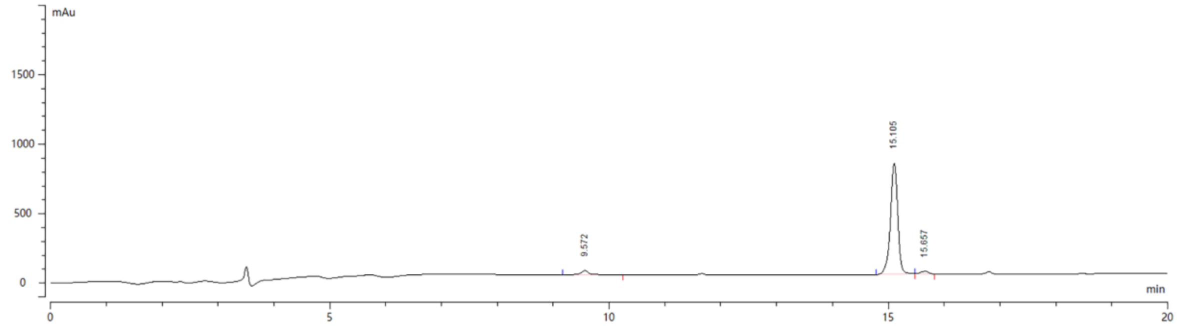

Compound	Ret. time (min)	Area (mAu*s)	Area (%)	Height (mAU)
N.A.	9.572	298.115	3.845	29.937
SegetalinJ	15.105	7291.813	94.046	798.809
N.A.	15.657	163.514	2.109	18.161

High-resolution MS of segetalin J

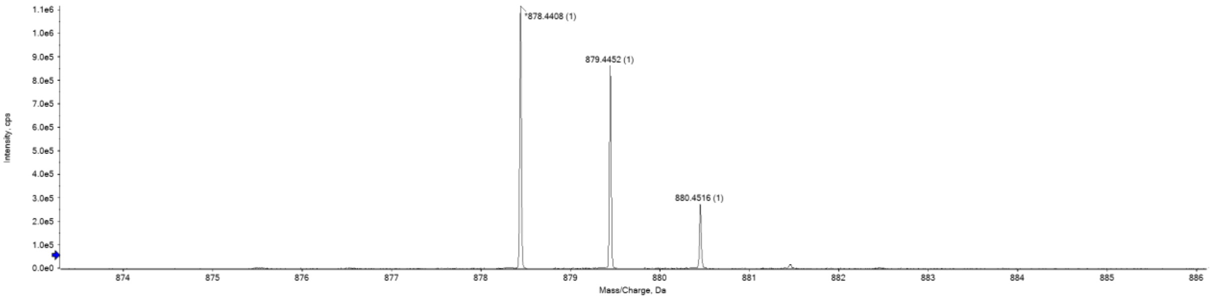

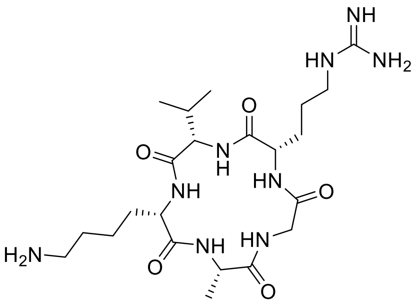

Segetalin K

***Cyclo*-[Gly-Arg-Val-Lys-Ala] (segetalin K)**

^{1}H NMR (600 MHz, D_2O) δ 4.13 (dd, *J* = 6.0 Hz, 12.0 Hz, 1H), 4.06 (dd, *J* = 6.0 Hz, 12.0 Hz, 1H), 3.97 (dd, *J* = 6.0 Hz, 9.6 Hz, 1H), 3.86-3.73 (m, 3H), 3.11-3.06 (m, 2H), 2.86-2.82 (m, 2H), 2.00-1.94 (m, 1H), 1.77-1.68 (m, 4H), 1.58-1.49 (m, 4H), 1.31-1.27 (m, 3H), 1.24 (d, *J* = 6.0 Hz, 3H), 0.81 (d, *J* = 4.4 Hz, 3H), 0.77 (d, *J* = 4.4 Hz, 3H).

^{13}C NMR (150 MHz, D_2O) δ 174.49, 174.09, 173.50, 173.36, 171.41, 156.68, 61.02, 55.01, 54.54, 50.03, 42.45, 40.28, 39.13, 29.44, 29.41, 27.05, 26.19, 24.54, 18.70, 17.99, 15.18. **HRMS (ESI)** $C_{22}H_{41}N_9O_5$ $[M+H]^+$ calcd:512.3304, found:512.3238.

Analytical HPLC of segetalin K

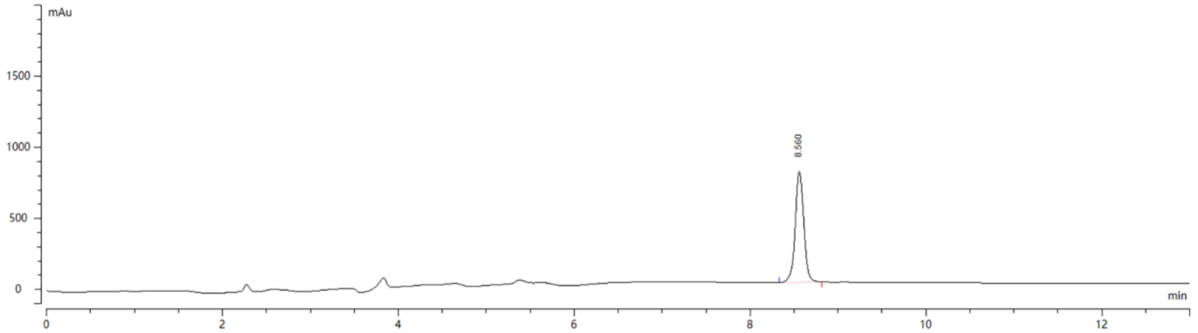

Compound	Ret. time (min)	Area (mAu*s)	Area (%)	Height (mAU)
SegetalinK	8.562	669.710	100.000	965.400

High-resolution Ms spectrum of segetalin K

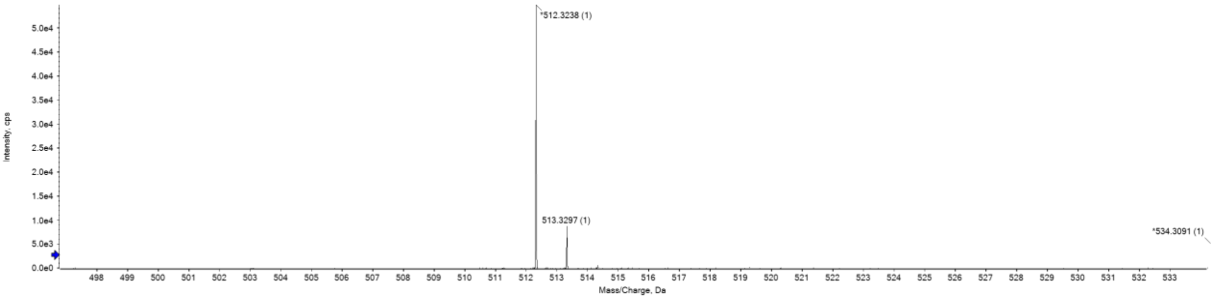

5. NMR spectra of products

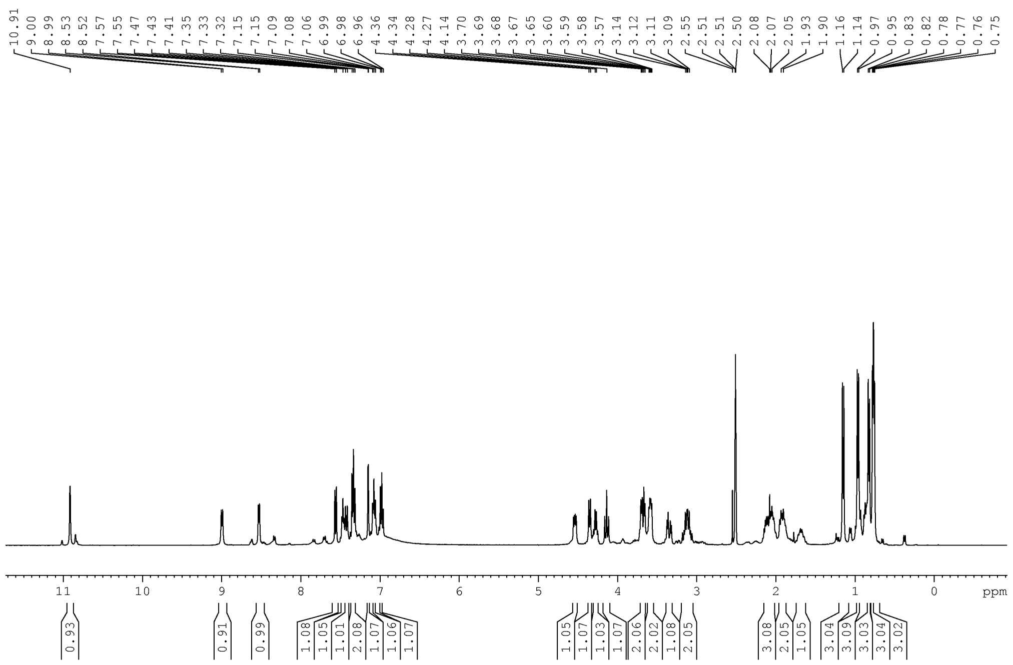

The ^{1}H NMR of **1** (DMSO-d_6, 400 MHz)

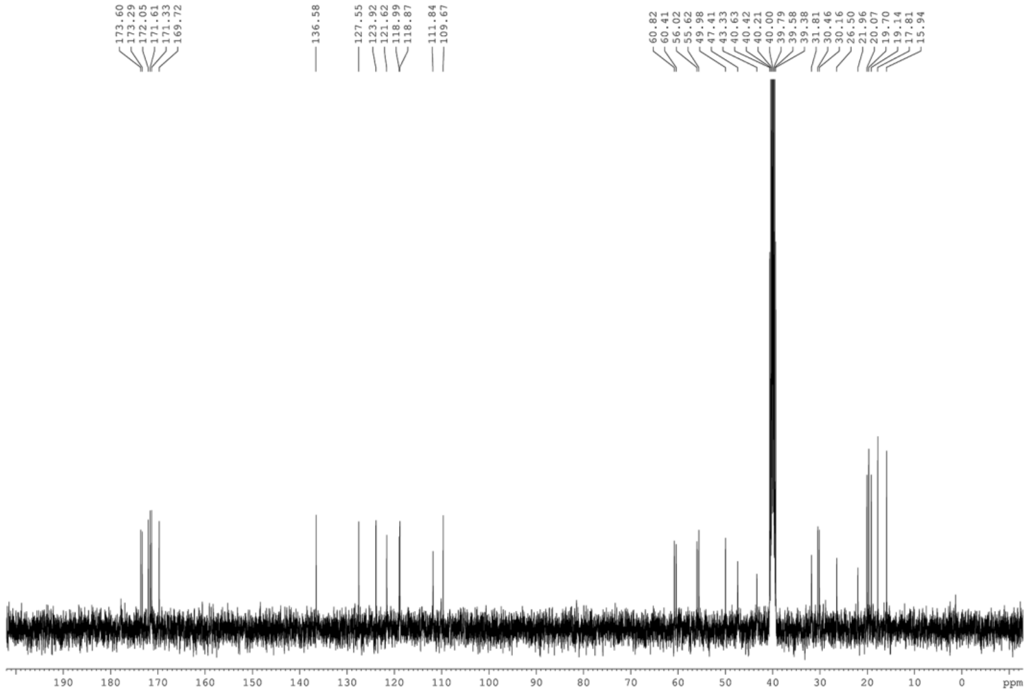

The ^{13}C NMR of **1** (DMSO-d_6, 100 MHz)

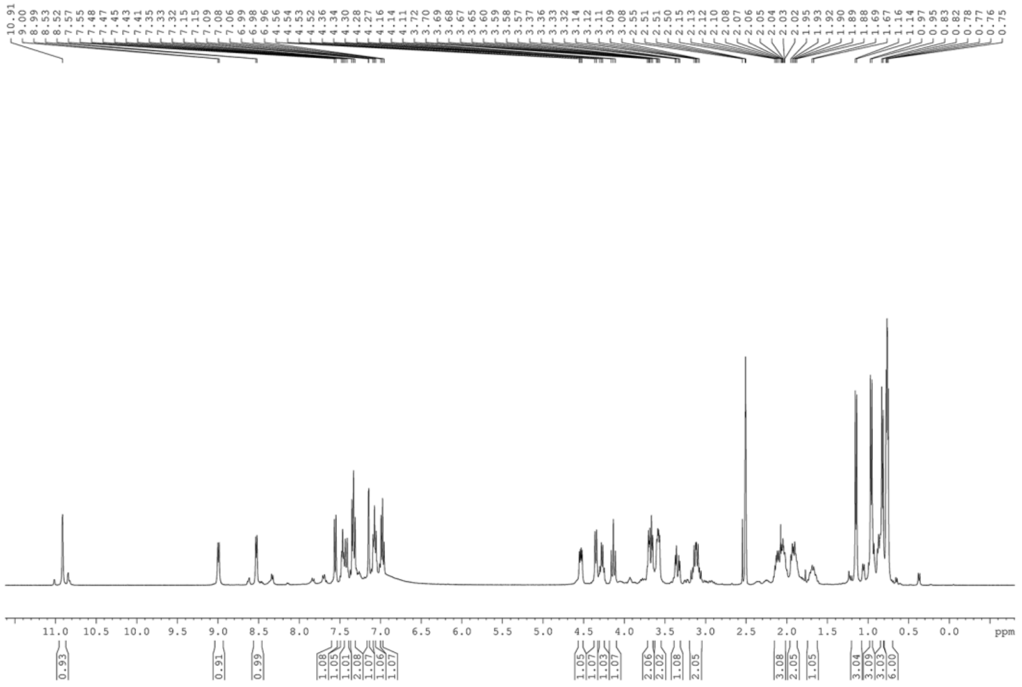

The ^{1}H NMR of **1** (DMSO-d_6, 400 MHz)

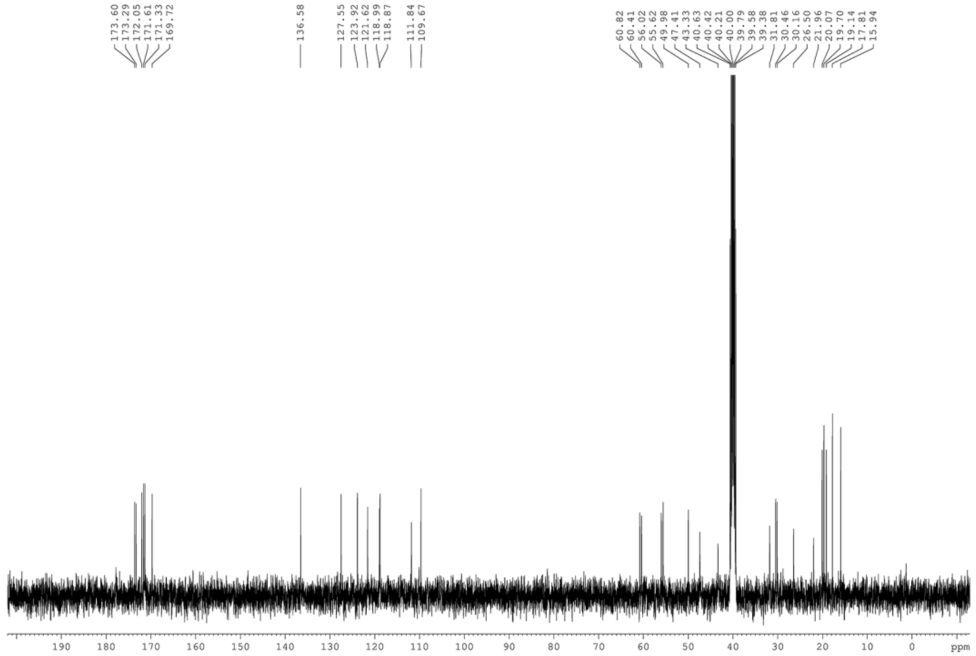

The ^{13}C NMR of **1** (DMSO-d_6, 100 MHz)

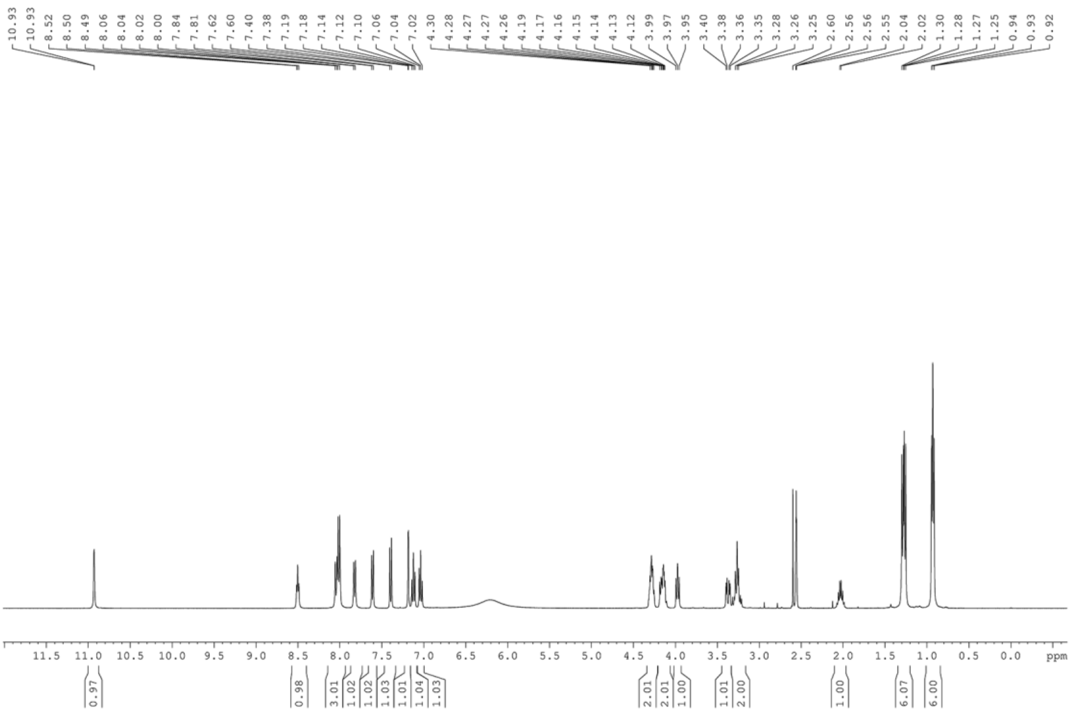

The ^{1}H NMR of **2** (DMSO-d_6, 400 MHz)

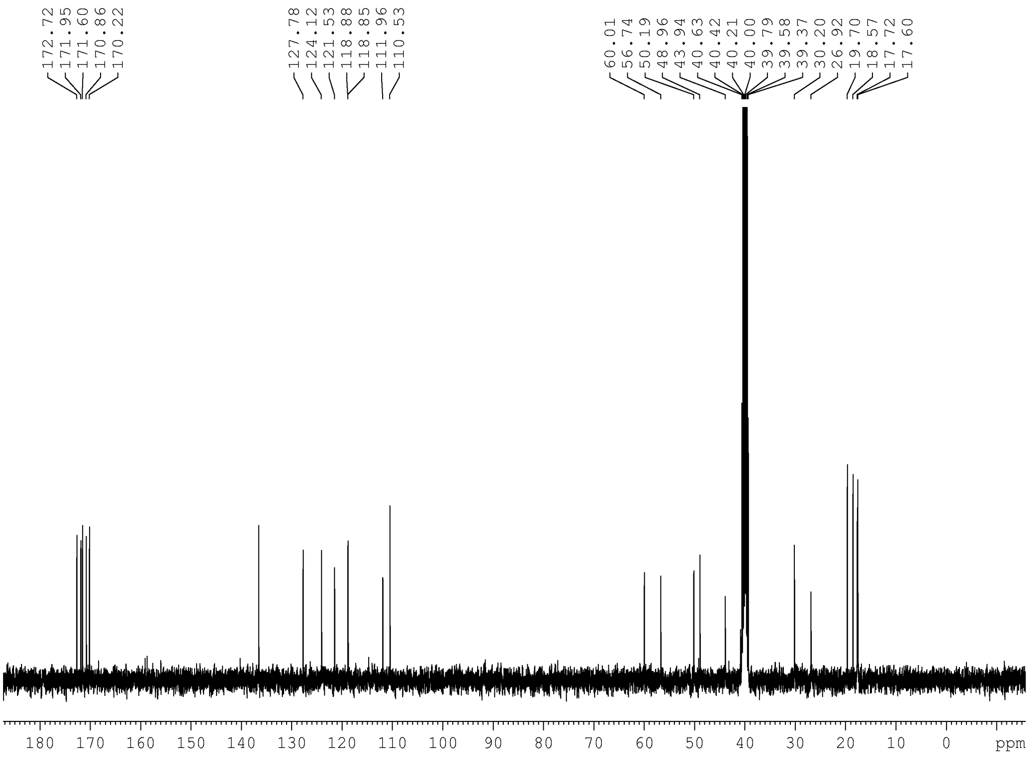

The ^{13}C NMR of **2** (DMSO-d_6, 100 MHz)

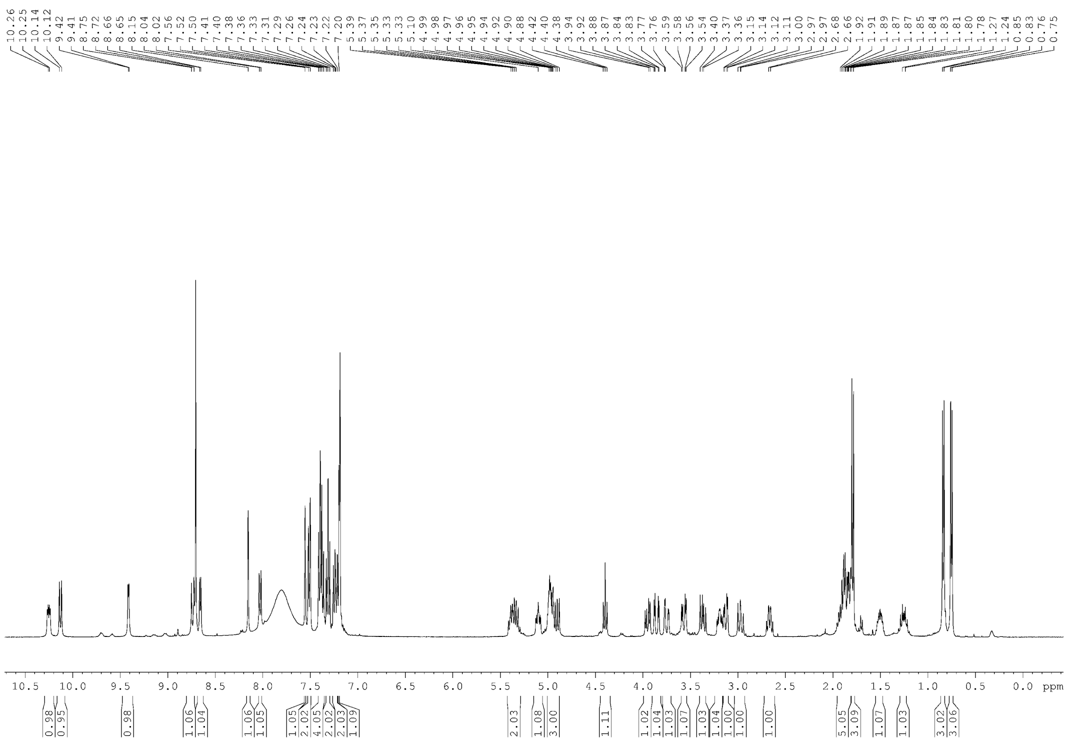

The ^{1}H NMR of **3** (pyridine-d_5, 400 MHz)

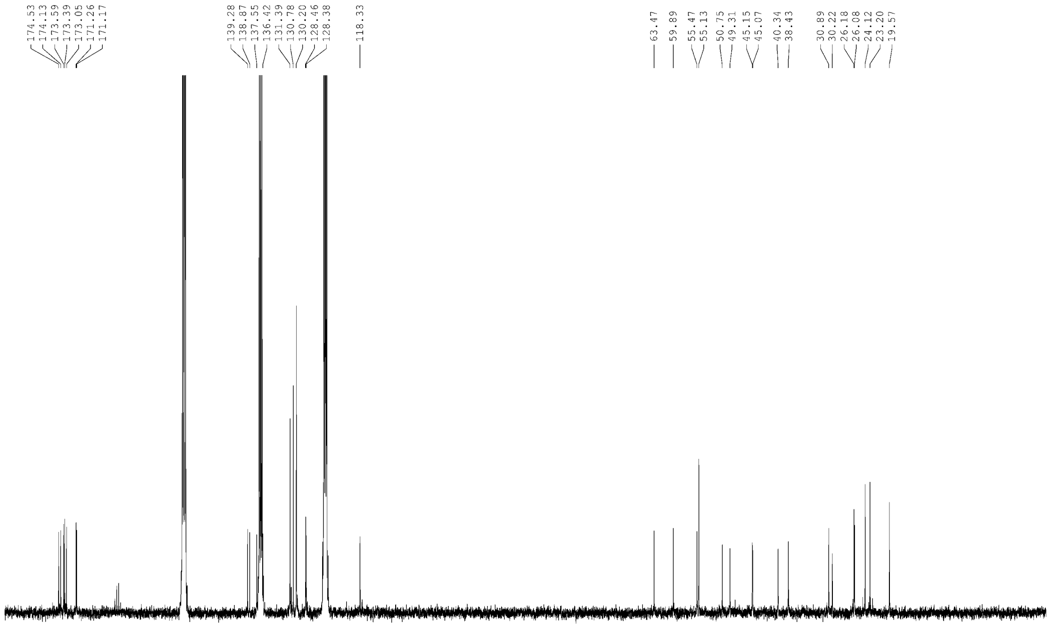

The ^{13}C NMR of **3** (pyridine-d_5, 100 MHz)

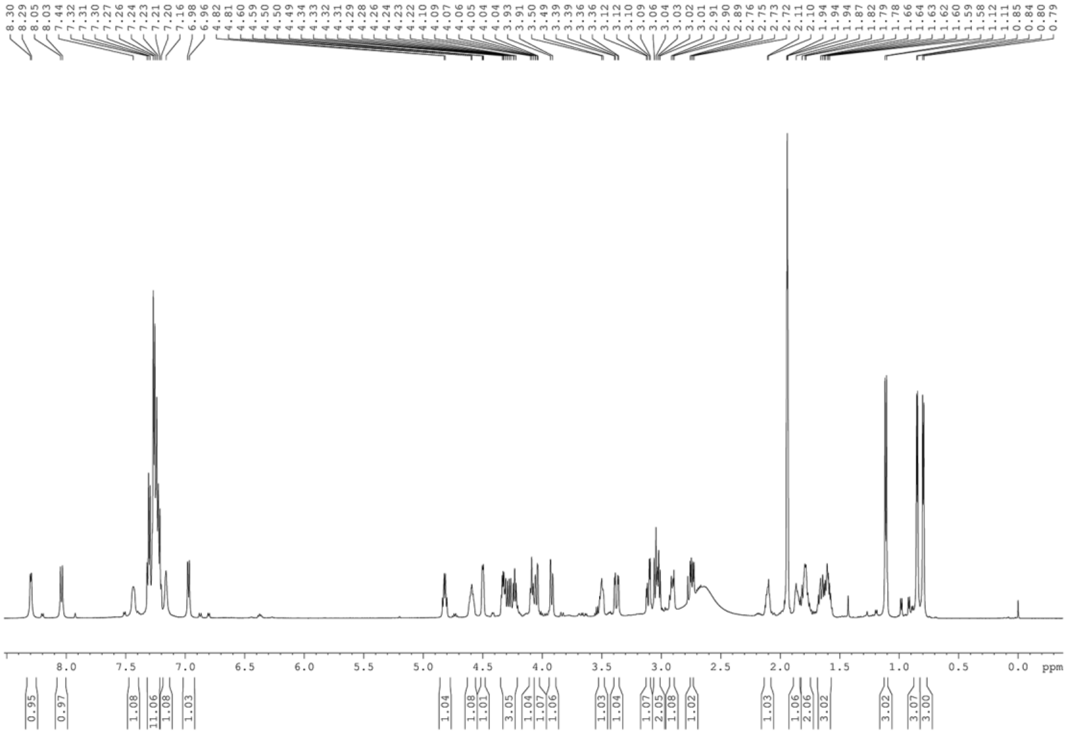

The ^{1}H NMR of **4** (CD_3CN, 600 MHz)

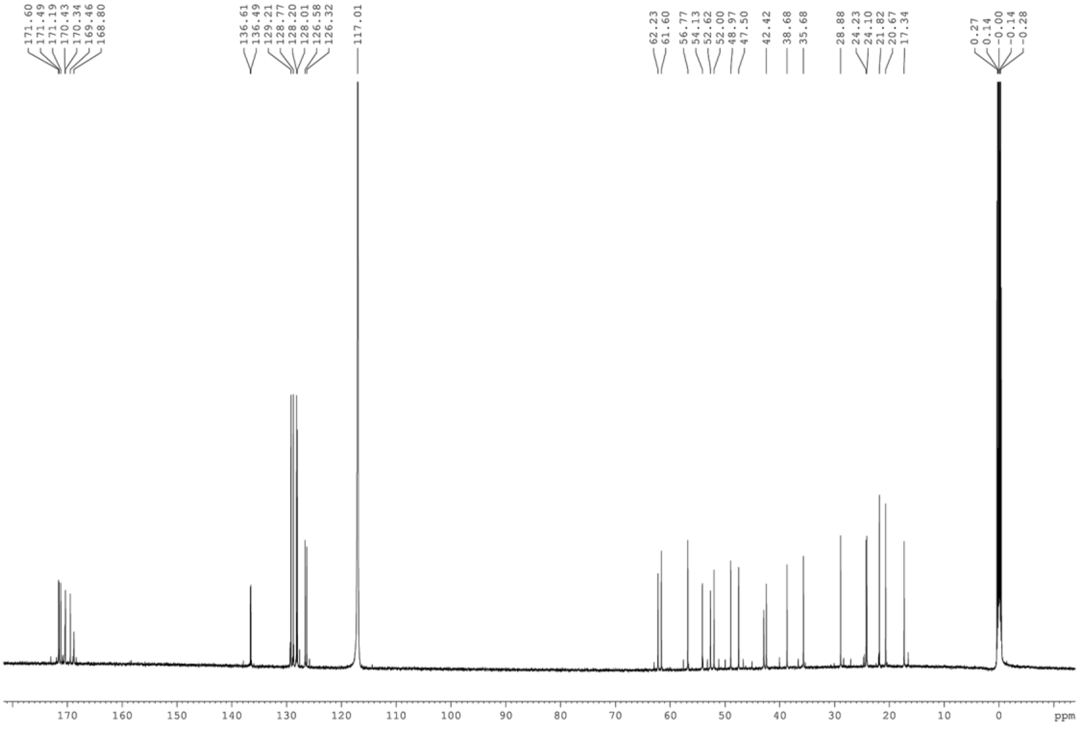

The ^{13}C NMR of **4** (CD_3CN, 150 MHz)

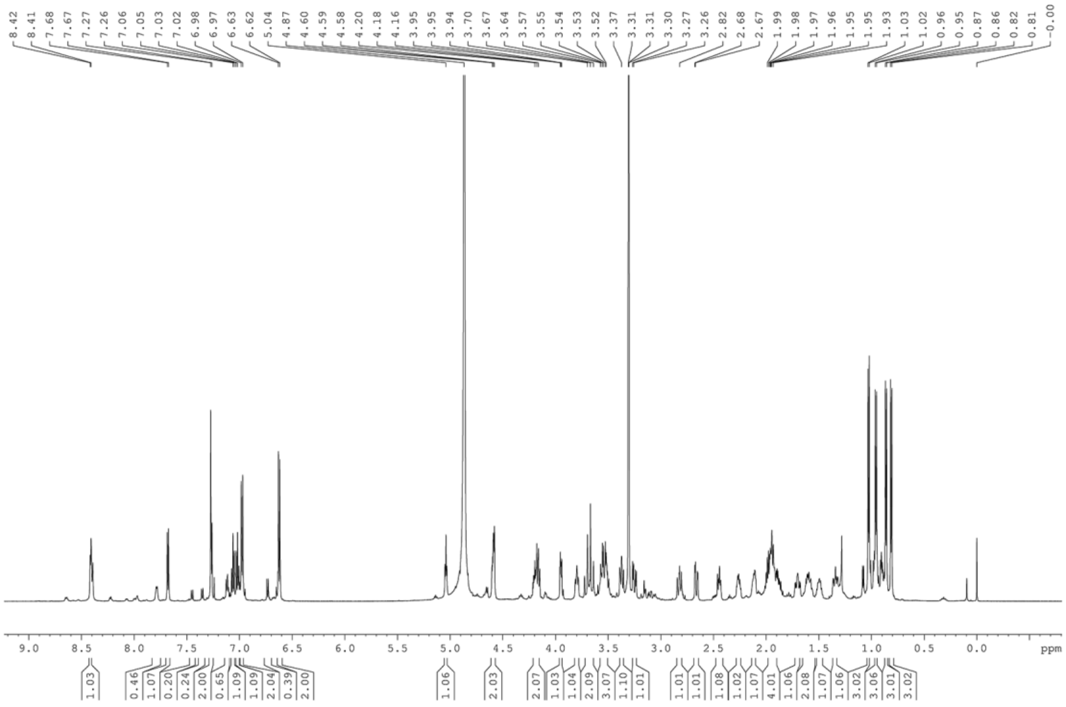

The ^{1}H NMR of **5** (CD_3OD, 600 MHz)

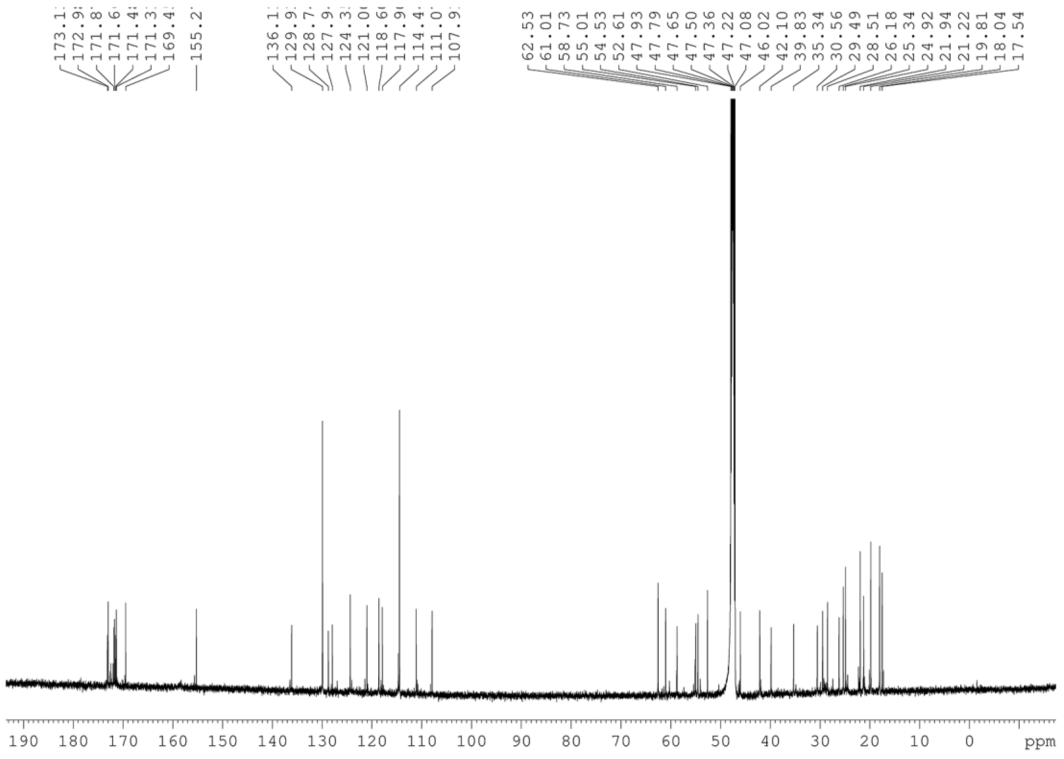

The ^{13}C NMR of **5** (CD_3OD, 150 MHz)

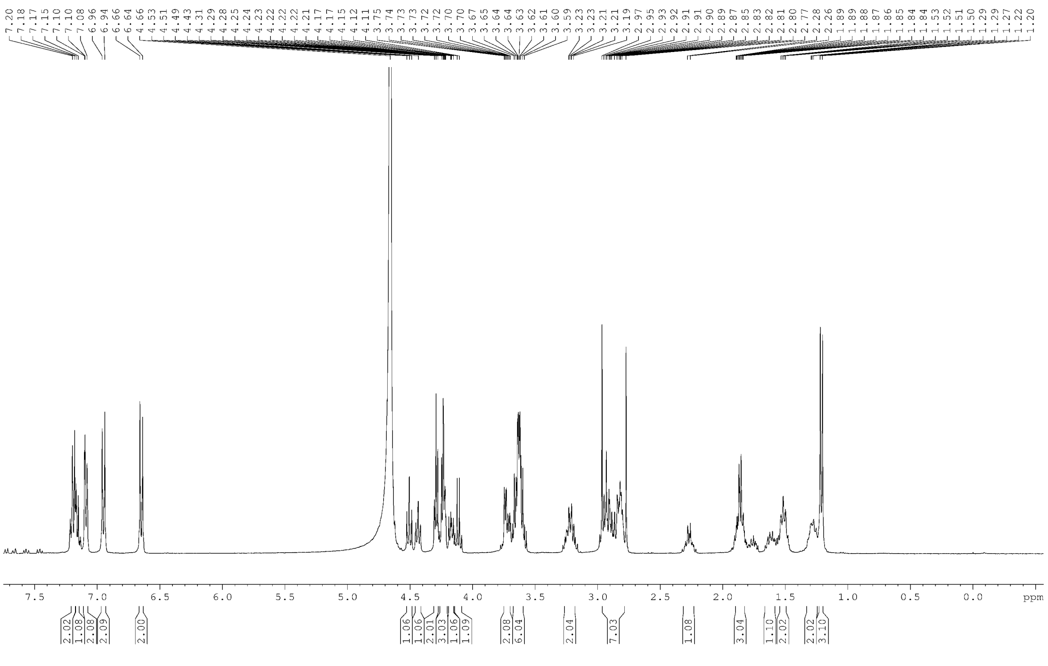

The ^{1}H NMR of **6** (D_20, 400 MHz)

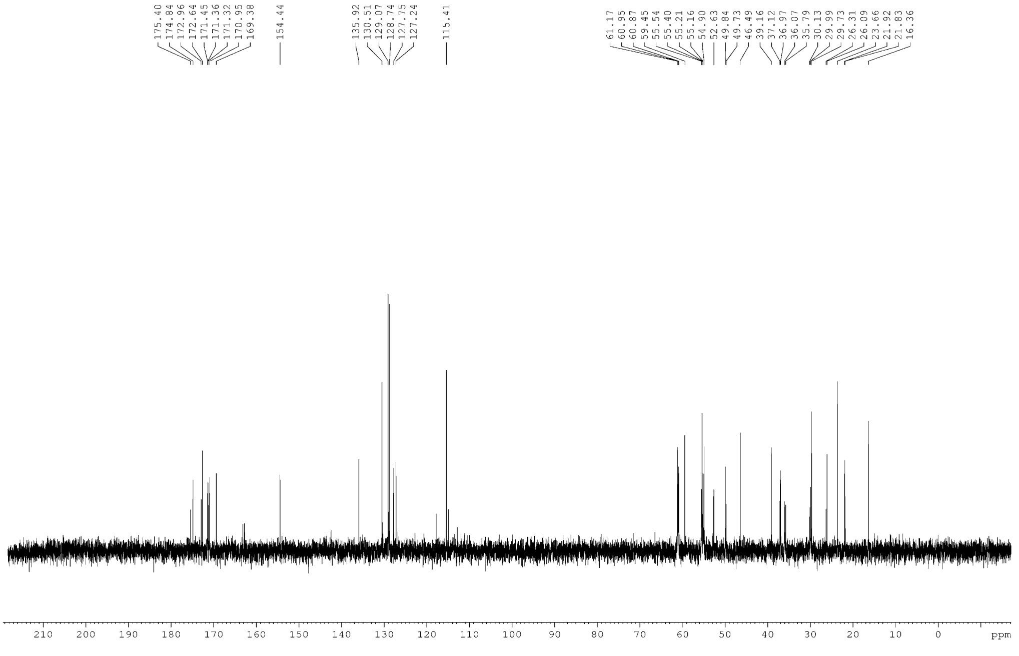

The ^{13}C NMR of **6** (D_2O, 100 MHz)

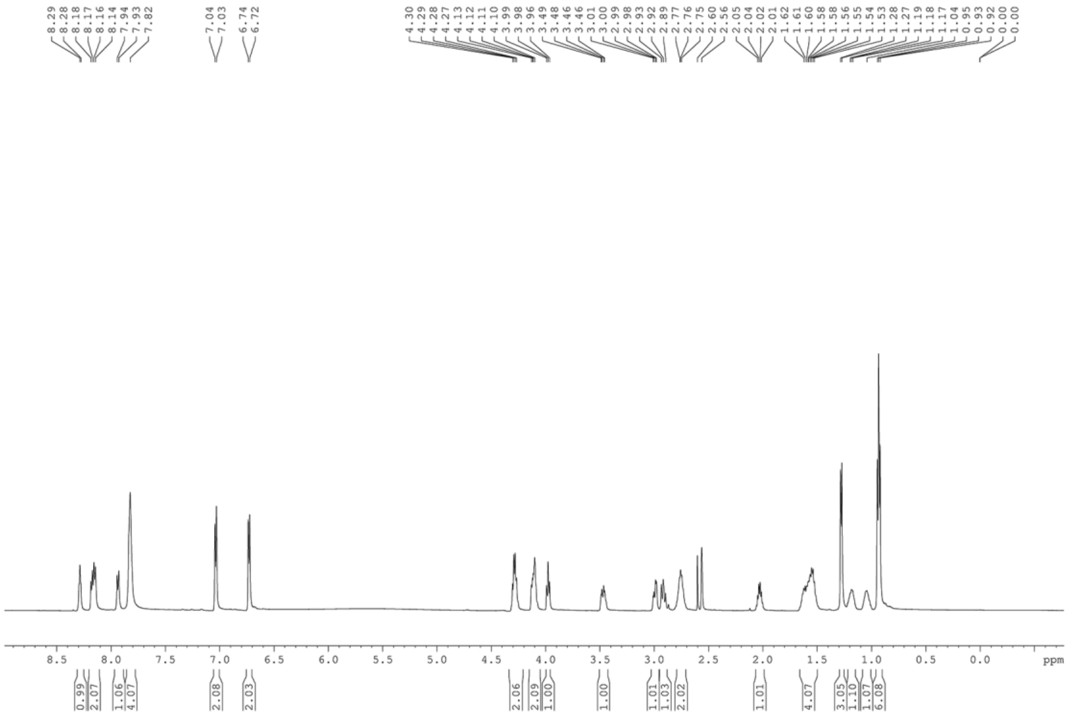

The ^{1}H NMR of **7** (D_2O, 600 MHz)

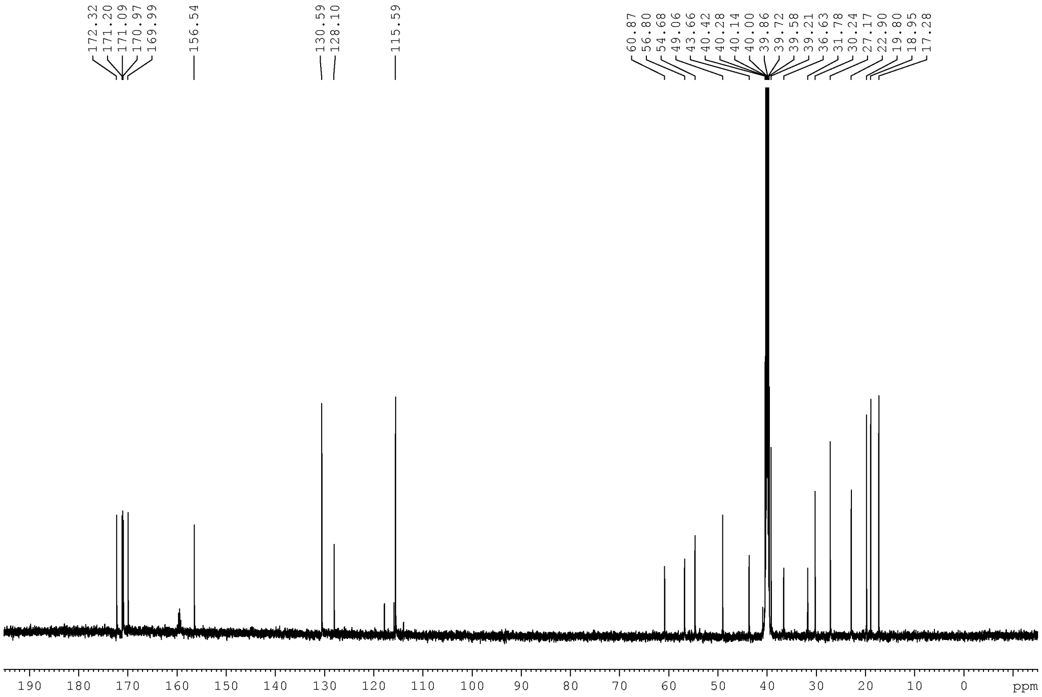

The ^{13}C NMR of **7** (D_2O, 150 MHz)

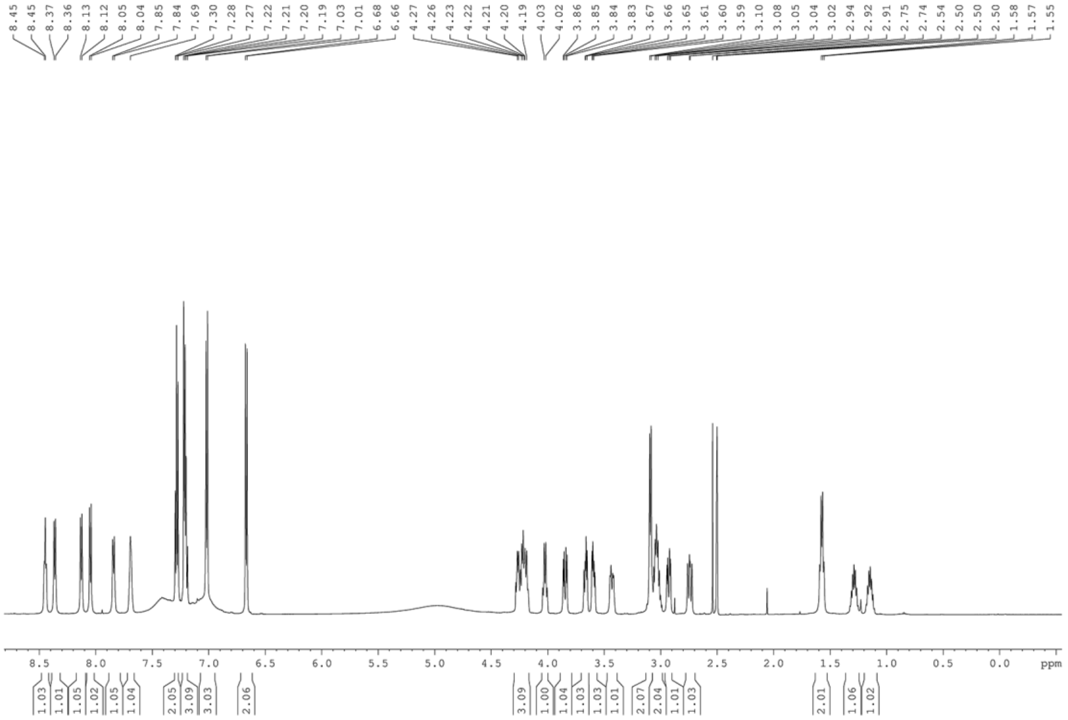

The ^{1}H NMR of **8** (D_2O, 600 MHz)

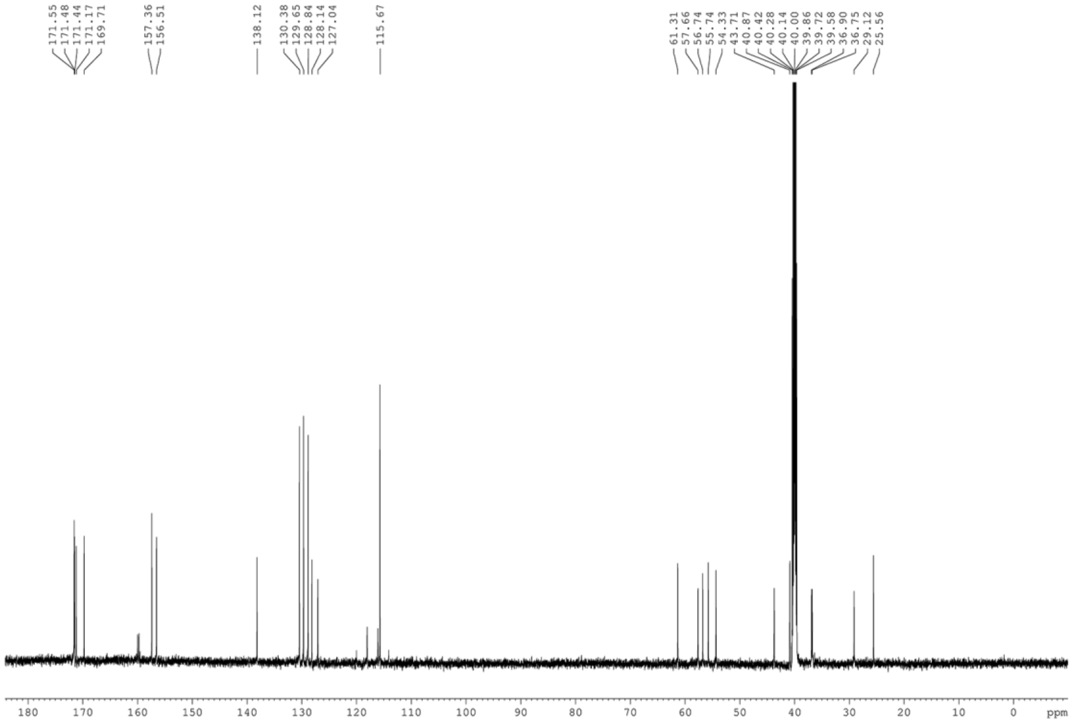

The ^{13}C NMR of **8** (D_2O, 150 MHz)

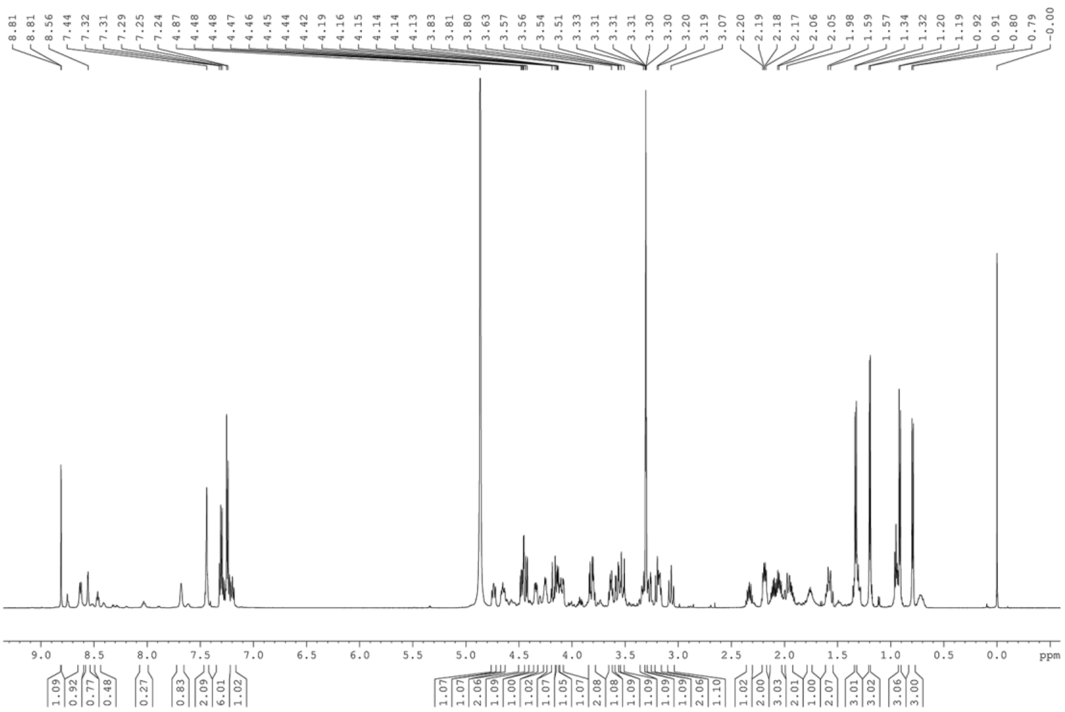

The ^{1}H NMR of **9** (CD_3OD, 600 MHz)

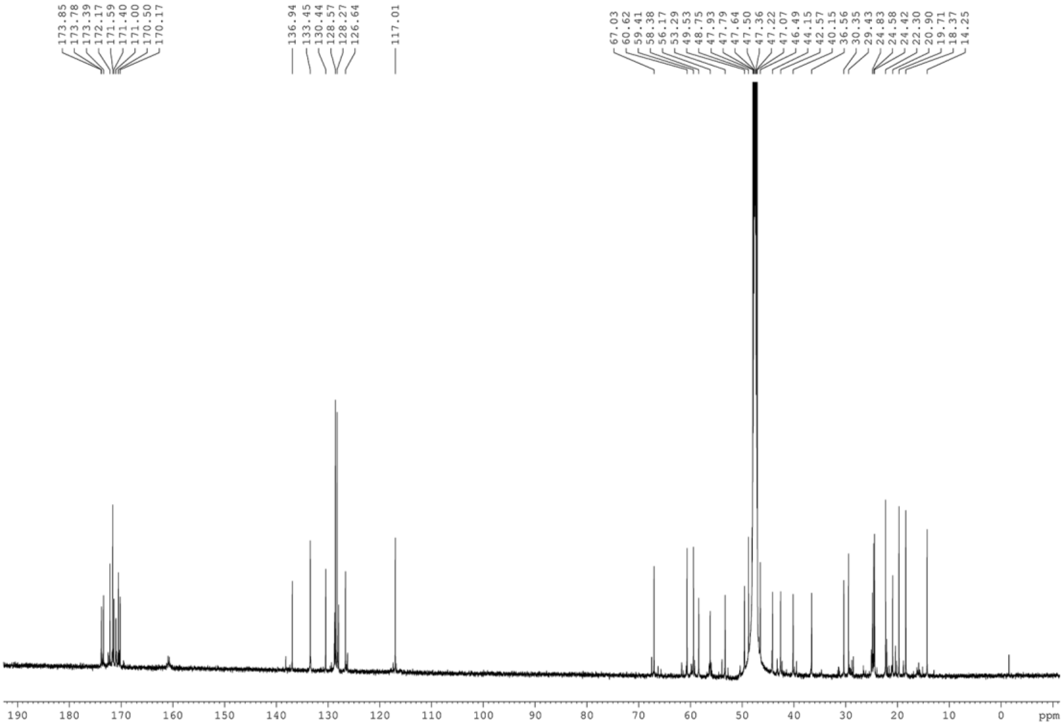

The ^{13}C NMR of **9** (CD_3OD, 150 MHz)

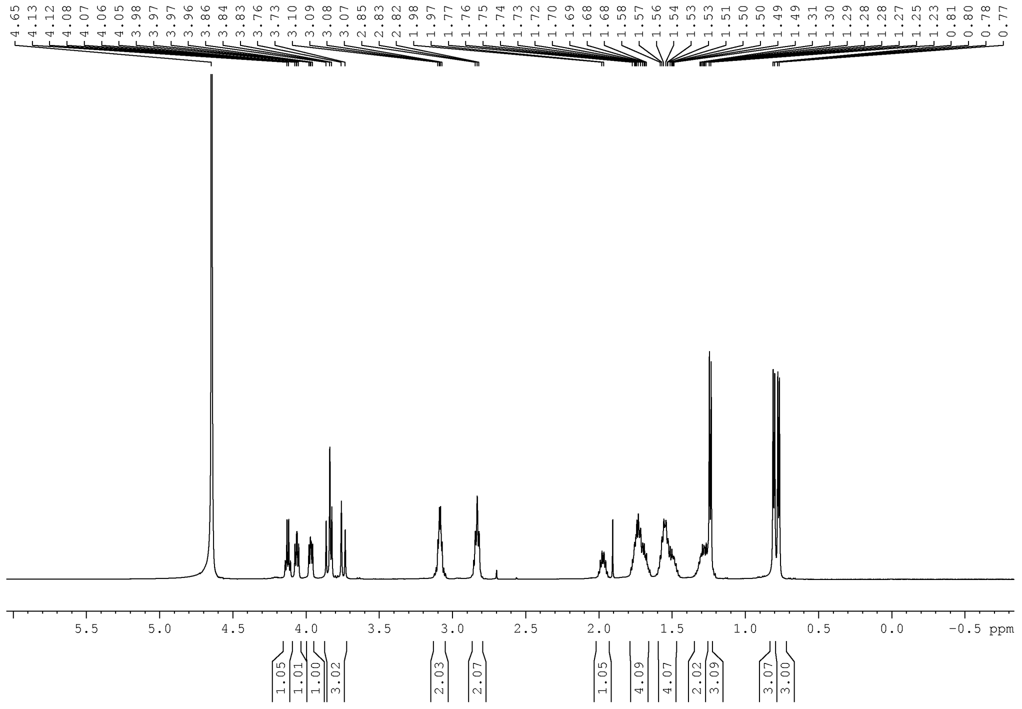

The ^{1}H NMR of **10** (D_2O, 600 MHz)

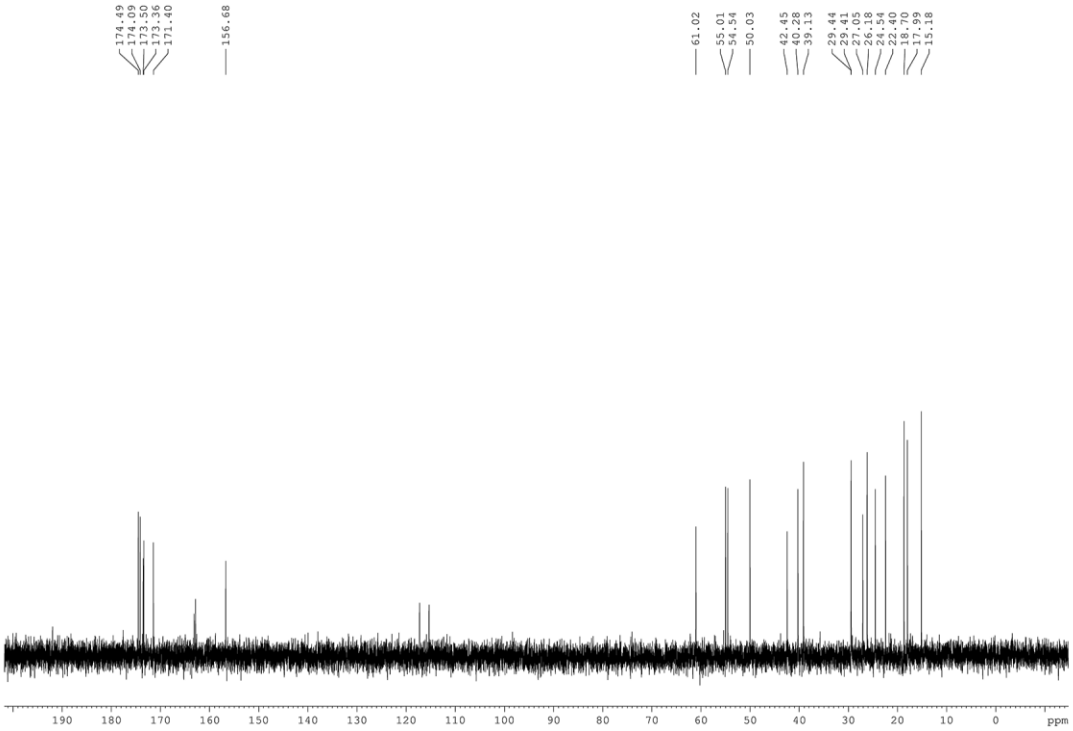

The ^{13}C NMR of **10** (D_2O, 150 MHz)
